# Whole exome sequencing study identifies novel rare and common Alzheimer’s-Associated variants involved in immune response and transcriptional regulation

**DOI:** 10.1038/s41380-018-0112-7

**Published:** 2018-08-14

**Authors:** Joshua C. Bis, Xueqiu Jian, Brian W. Kunkle, Yuning Chen, Kara L. Hamilton-Nelson, William S. Bush, William J. Salerno, Daniel Lancour, Yiyi Ma, Alan E. Renton, Edoardo Marcora, John J. Farrell, Yi Zhao, Liming Qu, Shahzad Ahmad, Najaf Amin, Philippe Amouyel, Gary W. Beecham, Jennifer E. Below, Dominique Campion, Camille Charbonnier, Jaeyoon Chung, Paul K. Crane, Carlos Cruchaga, L. Adrienne Cupples, Jean-François Dartigues, Stéphanie Debette, Jean-François Deleuze, Lucinda Fulton, Stacey B. Gabriel, Emmanuelle Genin, Richard A. Gibbs, Alison Goate, Benjamin Grenier-Boley, Namrata Gupta, Jonathan L. Haines, Aki S. Havulinna, Seppo Helisalmi, Mikko Hiltunen, Daniel P. Howrigan, M. Arfan Ikram, Jaakko Kaprio, Jan Konrad, Amanda Kuzma, Eric S. Lander, Mark Lathrop, Terho Lehtimäki, Honghuang Lin, Kari Mattila, Richard Mayeux, Donna M. Muzny, Waleed Nasser, Benjamin Neale, Kwangsik Nho, Gaël Nicolas, Devanshi Patel, Margaret A. Pericak-Vance, Markus Perola, Bruce M. Psaty, Olivier Quenez, Farid Rajabli, Richard Redon, Christiane Reitz, Anne M. Remes, Veikko Salomaa, Chloe Sarnowski, Helena Schmidt, Michael Schmidt, Reinhold Schmidt, Hilkka Soininen, Timothy A. Thornton, Giuseppe Tosto, Christophe Tzourio, Sven J. van der Lee, Cornelia M. van Duijn, Badri Vardarajan, Weixin Wang, Ellen Wijsman, Richard K. Wilson, Daniela Witten, Kim C. Worley, Xiaoling Zhang, Alzheimer’s Disease Sequencing Project, Celine Bellenguez, Jean-Charles Lambert, Mitja I. Kurki, Aarno Palotie, Mark Daly, Eric Boerwinkle, Kathryn L. Lunetta, Anita L. Destefano, Josée Dupuis, Eden R. Martin, Gerard D. Schellenberg, Sudha Seshadri, Adam C. Naj, Myriam Fornage, Lindsay A. Farrer, Alzheimer’s Disease Sequencing Project

**Affiliations:** 1 Department of Medicine (General Internal Medicine), University of Washington, Seattle, WA, USA; 2 Institute of Molecular Medicine, McGovern Medical School, University of Texas Health Science Center at Houston, Houston, TX, USA; 3 John P. Hussman Institute for Human Genomics, Miller School of Medicine, University of Miami, Miami, FL, USA; 4 Departments of Biostatistics, Boston University School of Public Health, Boston, MA, USA; 5 Case Western Reserve University, Cleveland Heights, OH, USA; 6 Human Genome Sequencing Center and Department of Molecular and Human Genetics, Baylor College of Medicine, Houston, TX, USA; 7 Department of Medicine (Biomedical Genetics), Boston University School of Medicine, Boston, MA, USA; 8 Department of Neuroscience and Ronald M Loeb Center for Alzheimer’s Disease, Icahn School of Medicine at Mount Sinai, New York, NY, USA; 9 Department of Genetics and Genomics Sciences, Icahn School of Medicine at Mount Sinai, New York, NY, USA; 10 University of Pennsylvania Perelman School of Medicine, Philadelphia, PA, USA; 11 Erasmus University Medical Center, Rotterdam, Netherlands; 12 Inserm, U1167, RID-AGE-Risk Factors and Molecular Determinants of Aging-Related Diseases, Lille, France; 13 Institut Pasteur de Lille, Lille, France; 14 University Lille, U1167-Excellence Laboratory LabEx DISTALZ, Lille, France; 15 Department of Medical Genetics, Vanderbilt University Medical Center, Nashville, TN, USA; 16 Department of Genetics and CNR-MAJ, Normandie Université, UNIROUEN, Inserm U1245 and Rouen University Hospital, F 76000, Normandy Centre for Genomic and Personalized Medicine, Rouen, France; 17 Department of Research, Centre Hospitalier du Rouvray, Sotteville-lès-, Rouen, France; 18 Department of Psychiatry, Washington University, St. Louis, MO, USA; 19 National Heart, Lung, and Blood Institute’s Framingham Heart Study, Framingham, MA, USA; 20 University of Bordeaux, Inserm, Bordeaux Population Health Research Center, team VINTAGE, UMR 1219, F-33000 Bordeaux, France; 21 Department of Neurology and Institute for Neurodegenerative Diseases, Bordeaux University Hospital, Memory Clinic, F-33000 Bordeaux, France; 22 Centre National de Recherche en Génomique Humaine, Institut François Jacob, Direction de le Recherche Fondamentale, CEA, Evry, France; 23 McDonnell Genome Institute, Washington University, St. Louis, MO, USA; 24 Broad Institute of MIT and Harvard, Cambridge, MA, USA; 25 Inserm UMR-1078, CHRU Brest, Université Brest, Brest, France; 26 Institute for Molecular Medicine Finland (FIMM), University of Helsinki, Helsinki, Finland; 27 National Institute for Health and Welfare, Helsinki, Finland; 28 Institute of Clinical Medicine - Neurology and Department of Neurology, University of Eastern Finland, Kuopio, Finland; 29 Institute of Biomedicine, University of Eastern Finland, Kuopio, Finland; 30 Program in Medical and Population Genetics and Genetic Analysis Platform, Stanley Center for Psychiatric Research, Broad Institute of MIT and Harvard, Cambridge, MA, USA; 31 Psychiatric & Neurodevelopmental Genetics Unit, Massachusetts General Hospital, Boston, MA, USA; 32 McGill University and Génome Québec Innovation Centre, Montréal, Canada; 33 Department of Clinical Chemistry, Fimlab Laboratories and Finnish Cardiovascular Research Center-Tampere, Faculty of Medicine and Life Sciences, University of Tampere, Tampere, Finland; 34 Department of Medicine (Computational Biomedicine), Boston University School of Medicine, Boston, MA, USA; 35 Columbia University, New York, NY, USA; 36 Indiana University School of Medicine, Indianapolis, IN, USA; 37 University of Tartu, Estonian Genome Center, Tartu, Estonia; 38 Department of Epidemiology, University of Washington, Seattle, WA, USA; 39 Department of Health Services, University of Washington, Seattle, WA, USA; 40 Kaiser Permanente Washington Health Research Institute, Seattle, WA, USA; 41 Inserm, CNRS, Univ. Nantes, CHU Nantes, l’institut du thorax, Nantes, France; 42 Unit of Clinical Neuroscience, Neurology, University of Oulu and Medical Research Center, Oulu University Hospital, Oulu, Finland; 43 Department of Neurology, Clinical Division of Neurogeriatrics, Medical University of Graz, Graz, Austria; 44 Department of Neurology, Kuopio University Hospital, Kuopio, Finland; 45 Department of Statistics, University of Washington, Seattle, WA, USA; 46 Department of Medicine (Medical Genetics), University of Washington, Seattle, WA, USA; 47 Department of Biostatistics, University of Washington, Seattle, WA, USA; 48 School of Public Health, University of Texas Health Science Center at Houston, Houston, TX, USA; 49 Departments of Neurology, Boston University School of Medicine, Boston, MA, USA; 50 Glenn Biggs Institute for Alzheimer’s and Neurodegenerative Diseases, University of Texas Health Sciences Center, San Antonio, TX, USA; 51 Department of Epidemiology, Boston University School of Public Health, Boston, MA, USA; 52 Department of Ophthalmology, Boston University School of Medicine, Boston, MA, USA

## Abstract

The Alzheimer’s Disease Sequencing Project (ADSP) undertook whole exome sequencing in 5,740 late-onset Alzheimer disease (AD) cases and 5,096 cognitively normal controls primarily of European ancestry (EA), among whom 218 cases and 177 controls were Caribbean Hispanic (CH). An age-, sex- and *APOE* based risk score and family history were used to select cases most likely to harbor novel AD risk variants and controls least likely to develop AD by age 85 years. We tested ~1.5 million single nucleotide variants (SNVs) and 50,000 insertion-deletion polymorphisms (indels) for association to AD, using multiple models considering individual variants as well as gene-based tests aggregating rare, predicted functional, and loss of function variants. Sixteen single variants and 19 genes that met criteria for significant or suggestive associations after multiple-testing correction were evaluated for replication in four independent samples; three with whole exome sequencing (2,778 cases, 7,262 controls) and one with genome-wide genotyping imputed to the Haplotype Reference Consortium panel (9,343 cases, 11,527 controls). The top findings in the discovery sample were also followed-up in the ADSP whole-genome sequenced family-based dataset (197 members of 42 EA families and 501 members of 157 CH families). We identified novel and predicted functional genetic variants in genes previously associated with AD. We also detected associations in three novel genes: *IGHG3* (p = 9.8 × 10^−7^), an immunoglobulin gene whose antibodies interact with β-amyloid, a long non-coding RNA *AC099552.4* (p = 1.2 × 10^−7^), and a zinc-finger protein *ZNF655* (gene- based p = 5.0 × 10−6). The latter two suggest an important role for transcriptional regulation in AD pathogenesis.

## Introduction

Genomic studies have revealed that late-onset Alzheimer disease (LOAD) is highly polygenic, with as many as 30 susceptibility loci identified through large-scale meta-analysis of genome-wide association studies (GWAS), targeted exome genotyping array, and several early whole exome sequencing (WES) studies [1–12]. Although AD susceptibility is highly heritable (*h*^*2*^ = 0.58–0.79) [13], much of its genetic architecture is still unknown and few rare variants have been detected thus far [3, 6, 7, 14–19]. Discovery of rare variants in genomic studies, even those with large sample sizes and examining highly heritable diseases, remains challenging due to statistical power limitations in detecting all but the most strongly associated variants (odds ratio (OR) > 1.5) [20–23]. The protein coding regions of the genome, or exome, are the best characterized and most conserved portions of the genome and the source of most variants identified to date that are responsible for Mendelian diseases [24]; thus, the exome is a more attractive and less expensive target for identifying rare variants of large effect on disease than the non-protein coding portion of the genome.

The Alzheimer’s Disease Sequencing Project (ADSP) was developed jointly by the National Institute on Aging (NIA) and National Human Genome Research Institute (NHGRI) in response to the National Alzheimer’s Project Act milestones (https://aspe.hhs.gov/national-alzheimers-project-act) to fight Alzheimer’s disease (AD) as an effort to analyze the genomes of well-characterized individuals with and without AD. To detect rare variants and genes associated with LOAD, we performed single-variant and gene-based analyses, including annotated loss-of-function analyses, on the ADSP Discovery Phase Case-Control WES dataset, and attempted to replicate associations in three independent WES datasets, a GWAS dataset containing single nucleotide variants (SNVs) that were imputed using the Haplotype Reference Consortium (HRC) [25] reference panel, and the ADSP family-based whole genome sequence dataset.

## Methods

### Sample selection and data preparation

Study participants were either European-American (EA) or Caribbean Hispanic (CH) ancestry and were sampled in two ways. To maximize contrast between cases and controls, and power to discover novel associations, the majority of participants were chosen using a risk score that included dosages of the *APOE* ε2/ε3/ε4 alleles, sex and either onset age (for cases) or age at last exam for controls (or pathology-based adjusted age at death for neuropathology control) [26]. All cases were at least 60 years old and met NINCDS-ADRDA criteria for possible, probable or definite AD based on clinical assessment, or had presence of AD (moderate or high likelihood) upon neuropathology examination. To maximize our ability to discover novel genetic associations, we chose cases whose AD risk score indicated that their disease was not well explained by age, sex, or dosages of the *APOE* ε2/ε3/ε4 alleles. Conversely, cognitively healthy controls were selected with the goal of identifying alleles associated with the increased risk of or protection from late-onset AD. At the time of last exam, all potential controls were at least 60 years old and were either judged to be cognitively normal or did not meet pathological criteria [27, 28] for AD following brain autopsy. Controls were selected for this study on the basis of the risk score indicating that they were the least likely to develop AD by age 85 years. Applying the risk score resulted in a sample that contained 2,220 AD cases (40*%*) and 752 controls (14*%*) who were ε4 heterozygotes and 161 AD cases (3*%*) and 17 controls (<1*%*) who were ε4 homozygotes.

In addition, we sampled a set of “enriched” cases from families having at least three affected members for whom the diagnosis of AD was verified by direct examination or review of cognitive testing data and medical records. Cases from early-onset AD families or families with a known *PSEN1, PSEN2*, or *APP* mutation were excluded. Within each family, we selected only one AD case, typically the member with the lowest *a priori* AD risk (based on the risk score defined above), provided this person had sufficient genomic DNA. In addition, because 172 of the “enriched” cases described above were of CH ancestry, we also selected a set of 171 age- and sex-matched cognitively normal CH participants to serve as controls. Participant characteristics are shown in Table 1A.

## Procedures

### Genotype calling and data processing

WES data were generated at the Broad Institute, the Baylor College of Medicine’s Human Genome Sequencing Center, and Washington University’s McDonnell Genome Institute. An effort was made to assign all samples from a study of origin to the same center and there was a relatively balanced number of cases and controls at each center. Genotypes for bi-allelic SNVs and insertion-deletion polymorphisms (indels) were called using ATLAS2 using version Ch37/ hg19 of the reference genome. A coordinated effort was implemented for centralized variant calling and quality control (QC) efforts in order to create one batch of data for analysis. Although there were differences in allele frequencies across sequencing centers for some variants, it was difficult to determine whether these represented technical artifacts of different capture kits, variability in genetic background among cohorts assembled for this study, or chance differences that will often occur for infrequent or rare variants. QC steps and methods for evaluating cryptic relatedness, population substructure, differential missingness, and variant annotation are described in the Supplementary Materials.

### Single-variant and gene-based association analyses

#### Statistical models & rationale for covariate adjustments

All models included adjustment for sequencing center and population substructure. Before conducting the primary analyses, we evaluated up to 20 PCs for association with AD status. Only ancestry-specific PCs that significantly associated with AD status (*P*<0.005) in at least one of the three adjustment models were included as covariates (EA subgroup: PC1, PC5, PC8, PC9, PC10, PC11, PC18; Hispanic analyses included PC1 and PC2). Because most participants for the discovery study were sampled to maximize differences in cases and controls based on age, sex, and *APOE* genotypes, we included only PCs and sequencing center in our base adjustment model (Model 0). We evaluated two other models that included several covariates in addition to those in the base model: Model 1 adjusted for sex and age at diagnosis or last follow-up; and Model 2 adjusted for *APOE* ε4 & ε2 dosages in addition to those included in Model 1. All analyses were performed separately by ancestry (EA and CH) using seqMeta (version 1.6) [29]; the primary analysis is an inverse variance-weighted meta-analysis of these two groups. Single variant tests were limited to variants with at least 10 copies of the minor allele across the total QCed sample (MAF~0.0005).

#### Gene-based association testing

Gene-based tests examine the aggregate effect of risk and protective variants within a region defined by gene annotations. We performed gene-based tests using SKAT-O, which optimally combines SKAT and burden tests [30]. For these analyses, the SKAT portion of the test included variants with a MAF≤0.05; the burden component aggregated variants with MAF≤0.01. The SKAT test used ‘Wu weights’, defined by a beta density function with pre-specified parameters a1 = 1 and a2 = 25, evaluated at the sample minor allele frequency. The SKAT-O statistic, a linear combination between a SKAT statistic (Q_skat_) and a burden statistic (Q_burden_) equal to (1-ρ) Q_skat_ + ρQ_burden_, was optimized across 11 values of ρ (0.1 increments), and calculation of the significance took into consideration the multiple values of ρ evaluated. In order to improve power by removing variants predicted to have a low functional impact on the translated protein, we filtered variants in each gene on the basis of annotated function as described in the Supplementary Materials. We performed SKAT-O testing for genes with at least two qualifying variants contributing to the test. The minimum number of aggregated alleles (i.e., cumulative minor allele count or cMAC) for a gene-based test was set at 10.

#### Statistical significance thresholds for discovery stage analyses

Within each analysis framework including individual variants and gene-based aggregation of variants evaluated under particular functional annotation criteria, suggestive associations (p < 1/ # tests) were selected for follow-up testing in independent samples and a Bonferroni-corrected threshold was used to define experiment wide statistical significance (p < 0.05 / # tests). We did not correct for the three models and meta-analyses of the combined results of the EA + CH populations because the results were highly correlated across the covariate adjustment strategies (Supplementary Figure S2).

#### Replication sample and analyses

Primary replication analyses for the SNVs / genes that we identified to be genome-wide significant or suggestive in any model were conducted in three independent WES datasets including CHARGE (612 cases and 1,836 controls), ADES-FR (1,142 cases and 1,104 controls) [31], and FinnAD (1,024 cases and 4,322 controls), as well as in the Alzheimer’s Disease Genetics Consortium (ADGC) HRC-imputed GWAS dataset (Table 1B, Supplementary Materials, Supplementary Table S1). The ADGC dataset included GWAS data on 9,343 cases and 11,527 cognitively normal elders from 32 datasets for whom genotypes were imputed using the Haplotype Reference Consortium (HRC) r1.1 reference panel (Supplementary Table S2) [25, 32]. CHARGE and ADGC participants selected for ADSP discovery analyses were not included in the replication study.

Because we included all available cases and controls in the replication datasets instead of applying the participant selection criteria used for the discovery sample to maximize difference in cases and controls, model 0 is not appropriate in replication studies. Hence, single variant tests and gene-based SKAT-O tests were performed using seqMeta for models 1 and 2 only. Meta-analysis of summarized results from the four samples was performed using seqMeta. We also performed a meta-analysis of results across the ADSP discovery and four replication cohorts for findings that were at least “suggestive” (p < 1 / genes or variants) in the discovery phase. In addition to models 1 and 2, we conducted a meta-analysis of results obtained using model 0 in the ADSP discovery data and model 1 in the replication cohorts to verify our findings in ADSP model 0. Because the ADSP discovery dataset includes CH participants and all replication cohorts consist of EA participants only, we performed the meta-analysis with and without CH participants in ADSP. We considered any variants or genes with two-stage meta-analysis p-values < 0.05 / # tests to be significant per the recommendation by Skol et al. that joint analysis is more efficient than replication-based analysis for two-stage genome-wide association studies [33]. We acknowledge, however, that additional replication in independent samples is required.

The top findings in the discovery sample were also followed-up in the ADSP whole-genome sequenced (WGS) family-based dataset [34, 35]. This dataset includes 197 individuals sequenced in 42 EA families and 351 individuals in 67 CH families. Additional follow-up was also performed using WGS information from 150 members of another 48 CH families. Individual variants were assessed by examining their co-segregation with AD status within families. Gene-based association tests were performed using the FSKAT software [36].

#### Analysis of variants at previously established AD loci

To identify a set of variants related to AD risk in loci previously associated with AD, we compiled a list of genes containing variants with significant or suggestive associations (p < 1 × 10^−3^) in either the published IGAP or UKBB AD GWAS meta-analyses [9, 37]. Because many signal variants from GWAS are in intergenic regions, we used a combination of BEDOPS [38] and BEDTools [39] operations to enlarge the genomic coordinates of these associated variants by 50 Kb on each side, merging adjacent regions that were overlapping and/or book-ended. Of the resulting genomic regions, segments greater than 100 kb were retained, shortened by 50 kb on each side, merged if separated by 200 kb or less, and utilized to find overlapping protein-coding genes, with gene boundaries as defined in version 19 of the GENCODE gene set [40] and a 50 kb buffer on each side. These parameters and sequence of operations were chosen because they resulted in an algorithm that satisfactorily captured the genomic interval of the association landscape at each locus, as confirmed by visual inspection of LocusZoom regional plots [41]. We queried variant and gene level association statistics for the resulting list of 299 putatively associated AD genes.

## Results

### Description of study samples after QC and filtering

After exclusions, 10,836 participants were available for analysis (5,740 cases; 5,096 controls). This included 218 CH cases and 177 CH controls. The study included more women than men, and, due largely to the selection criteria, cases were younger on average than controls and were more likely to carry one copy of the *APOE* ε4 allele. In total, the data included 1,524,414 bi-allelic SNVs or short indels. Most variants were rare, with 1,493,926 (98*%*) of variants having minor allele frequency of less than 5*%* and 160,898 (11*%*) having a minor allele count (MAC) of at least 10 copies.

### Single-variant SNV and short indels association analysis

**Fig. 1:**
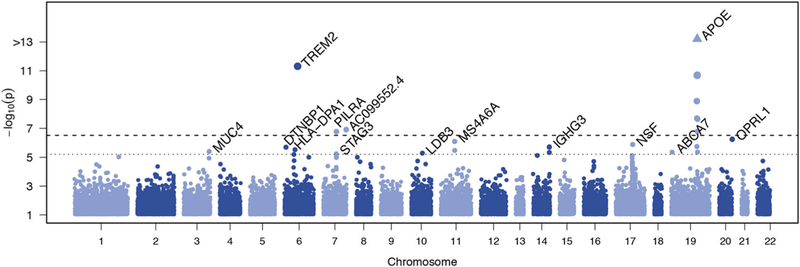
Manhattan plot showing genome-wide association results for individual common variants. The plot shows the p-values from the Discovery meta-analysis against their genomic position for association with AD. Only variants with a combined minor allele count of ≥ 10 were included; the minimum p-value from the three adjustment models for either the meta-analysis, European Ancestry (EA), or Caribbean Hispanic (CH) is plotted for each variant. Genes containing the variant are indicated above points that surpassed our significance threshold for follow-up. The dotted line indicates the threshold for follow-up, p < 6.1 × 10^−6^, corresponding to (1 / #variants) tested. The dashed line indicates the threshold for exome-wide significance, p < 3.1 × 10^−7^, corresponding to (0.05 / #variants tested)

We performed single variant analyses for the 160,898 variants with a combined minor allele count of at least 10 copies across all participants (Supplementary Figure S3). Genomic inflation was moderate (λ < 1.1 in all models) (Supplementary Figures S4–S7). Single variant association testing identified three variants at an exome-wide significance level (p < 3.1 × 10^−7^) and 14 variants at the suggestive threshold (p < 6.1 × 10^−6^) outside of the *APOE* region (Table 2, Fig. 1, Supplementary Table S3). The significant associations included the rare missense R47H variant in *TREM2* (rs75932628, p = 4.8 × 10^−12^), a common variant in *PILRA* (rs2405442, p = 1.7 × 10^−7^), and a novel rare variant in the long non-coding RNA *AC099552.4* (7:154,988,675, p = 1.2 × 10^−7^). These results were attenuated when including age, sex, and *APOE* ε2/ε3/ε4 allele as covariates.

### Gene-based association analysis combining SNVs and indels

**Fig. 2:**
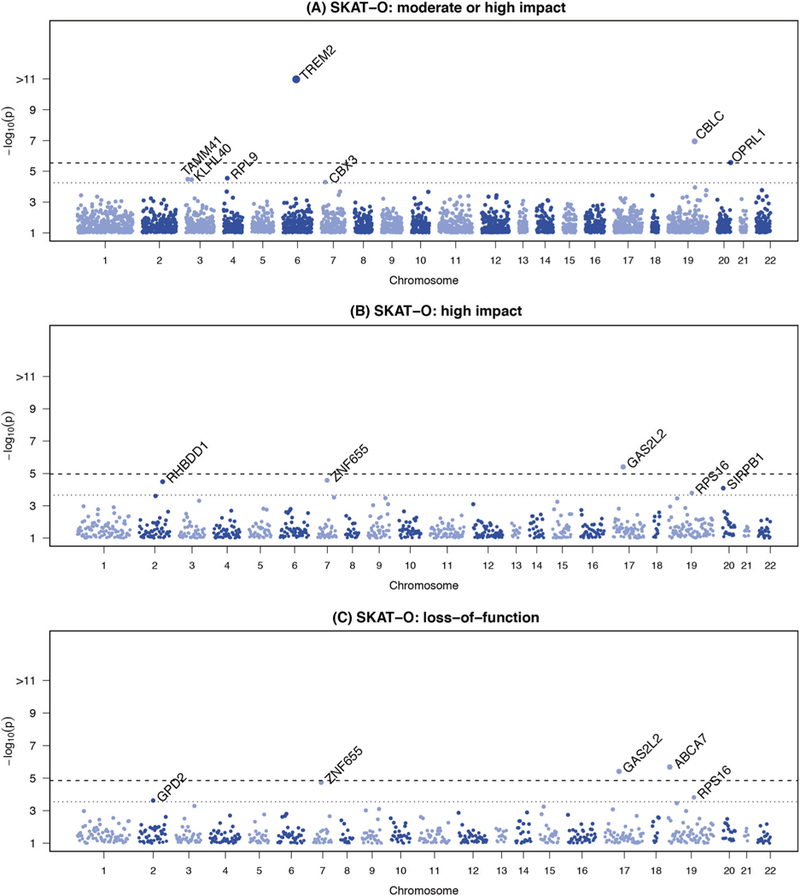
Manhattan plots showing exome-wide association results for gene-based tests of rare functional variants. The plots show the gene-based p-values from the Discovery meta-analysis against their geno-mic position for association with AD. Each point represents a p-value from SKAT-O test aggregating rare variants (MAF < 5*%*), by gene, on the basis of predicted functional impact. Only genes with a cumulative minor allele count of ≥ 10 were included; the minimum p-value from the three adjustment models for either the meta-analysis, European Ancestry (EA), or Caribbean Hispanic (CH) is plotted for each variant. Genes are indicated above points that surpassed our significance threshold for follow-up in tests aggregating only (**a**) moderate or high impact variants, (**b**) high impact variants; (**c**) loss-of-function variants. In each plot, the dotted line indicates the threshold for follow-up: (**a**) p<5.5 × 10^−5^, (**b**) p<6.3 × 10^−5^, (**c**) p<2.8 × 10^−4^, each corresponding to 1 / # genes tested. The dashed line indicates the threshold for exome-wide significance: (**a**) p<2.7 × 10^−6^, (**b**) p<3.1 × 10^−6^, (**c**) p < 1.4 × 10^−5^, each corresponding to 0.05 / # genes tested

We aggregated 918,053 variants with a combined MAF < 0.05 and annotated as high or moderate impact into gene-based tests using SKAT-O. This corresponds to 17,613 genes with more than one variant and a cumulative minor allele count (cMAC) of at least 10 copies. Applying more stringent filtering, we limited to variants annotated as high impact; aggregating 42,502 rare or uncommon (MAF < 0.05) variants into 4,634 genes (again, limiting to genes with >1 variant and a cMAC ≥ 10). For the purposes of identifying novel associations, we considered all genes or variants within 250kb of *APOE* as part of the *APOE locus*. Three known genes (*ABCA7, TREM2* and *CBLC* in the *APOE* region) and two novel genes (*OPRL1* and *GAS2L2*) achieved exome-wide statistical significance for their respective tests in the discovery analyses (Table 3, Fig. 2). Four additional genes (*ZNF655, RHBDD1, SIRPB1*, and *RPS16*) reached suggestive significance across the nine models (Fig. 2, Supplementary Table S4). Analyses filtered to include only variants with CADD scores ≥ 15 or ≥ 20 produced most of the same top-ranked results as the VEP gene-based results (Supplementary Table S5), noting that the overall VEP High/Moderate and CADD≥15 results, as well as the VEP High and CADD≥20 results, are only moderately correlated (Spearman rank correlation r = 0.51) (Supplementary Figure S8). Three novel genes (*CACNB3, HHIP-AS1,* and *RP11–68L1.1*) were exome-wide statistically significant in the CH group in analyses restricted to variants with CADD scores ≥ 20 (Supplementary Table S5), however these are likely false positives because in each instance the result is accounted for by a single variant that was observed in one person only.

### Loss-of-function (LOF) association analysis

**Fig. 3:**
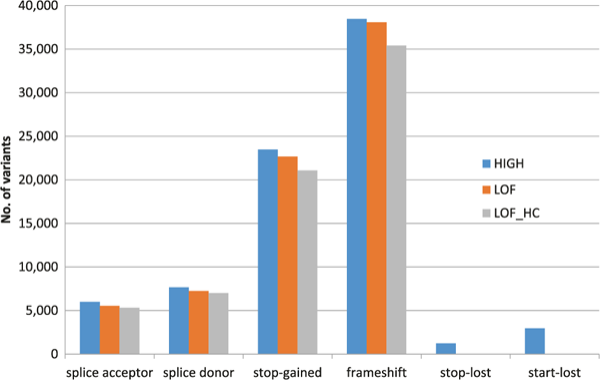
Distribution of high impact, LoF and high-confidence LoF variants grouped by predicted consequence

Among 78,529 unfiltered high impact variants, 72,694 were annotated as LoF and 68,121 were further deemed as high-confidence, most of which were frameshift and stop-gained (Fig. 3). As expected, over 90*%* of these high-confidence LoF variants were singletons (53,120, 78*%*), doubletons (6,579, 10*%*), or tripletons (2,222, 3*%*), and most of these were observed in European Americans only. Association analysis of 2,378 high-confidence LoF variants with MAC ≥ 10 with adjustment for sequencing center and PCs revealed one Bonferroni corrected significant p < 2.1 × 10^−5^) variant, a previously reported frameshift deletion in *ABCA7* (Table 3) [42]. Gene-based analysis of 32,863 high-confidence LoF variants with MAF ≤ 5*%* mapping to 3,558 genes with at least two variants and cMAC ≥ 10 (Supplementary Table S4) also showed that *ABCA7* with adjustment for sequencing center and PCs (Model 0) and *GAS2L2* with adjustment for sequencing center, PCs, sex, age, and *APOE* genotype (Model 2) reached experiment-wide significance threshold p < 1.4 × 10^−5^).

### Replication analysis

Of the 16 single variants outside the *APOE* region tested in the replication samples (Table 2, Supplementary Table S3), the *TREM2* R47H mutation and four variants in three other previously known genes (one missense and one synonymous variant in *MS4A6A*, a synonymous variant in *PILRA*, and a missense variant in CR1) were significantly associated with AD in the combined discovery and replication analysis (Table 2). Associations with two variants in a novel gene *STAG3* (rs1043915, p = 5.5 × 10^−6^) were also replicated and significantly associated with AD. We were unable to assess replication with the novel *AC099552.4* variant because it was not observed or imputed in the replication datasets. One of the *IGHG3* variants (rs12890621) showed borderline evidence for association in models 1 and 2 (p = 0.085 and 0.075, respectively), and evidence for association was strengthened to near exome-wide significance (p = 9.8 × 10^−7^) in the combined discovery and replication sample.

In total, 19 genes across the nine models were significantly or suggestively associated with AD and were tested in the replication stage (Supplementary Table S4). Gene-based tests including high or moderate impact variants showed evidence for replication and reached genome-wide significance in the combined discovery and replication analysis for three genes:*TREM2* and two genes in the *APOE* region (*CBLC* and *BCAM*) (Table 3, Supplementary Table S6). The association with *GAS2L2*, the potential novel gene identified in a model 2 SKAT-O test with high impact SNVs in the discovery sample, was slightly above the nominal significance level (p = 0.051 and p = 0.067, respectively, in models 1 and 2) in meta-analysis across four replication cohorts. However, this association was only nominally significant in the meta-analysis combining the discovery and replication cohorts (p = 0.029 for model 2).

In gene-based tests including only high impact SNVs, the known AD risk gene *ABCA7* and the potential novel gene *ZNF655* reached the nominal p-value of 0.05 in meta-analysis with replication cohorts as well as in a meta-analysis of discovery and replication samples (Table 3, Supplementary Table S6). These two genes were also nominally significant in SKAT-O tests, limited to high impact variants (*ZNF655*: p = 7.9 × 10^−6^; *ABCA7*: p = 6.2 × 10^−5^) and LOF variants (*ZNF655*: p = 5.0 × 10^−6^).

Because *PILRA*, a previously established AD gene [43] is proximate to *STAG3* (159 kb) and *ZNF655* (797 kb), we performed conditional analysis in the discovery sample to determine whether these novel association signals are independent. These analyses demonstrated that the association with multiple rare variants *ZNF655* in the gene-based test is distinct from those with common variants in *PILRA* (p = 1.08 × 10^−4^) and *STAG3* (p = 7.75 × 10^−5^). In a model containing both *PILRA* and *STAG3* variants the association with *PILRA* remains significant (p = 0.011) but the association with *STAG3* does not (p = 0.21) (Supplementary Table S7).

### Follow-up in ADSP family-based data

We also followed-up the significant and suggestive single-variant and gene-based results from the discovery stage in the ADSP whole-genome sequenced (WGS) family-based data- set. A rare missense variant (rs61756195, MAF = 0.001) in *STAG3* segregated with disease in three CH families and trended toward association in the case-control study (p = 0.052) (Supplementary Table S8). Gene-based testing identified a nominal association for *GAS2L2* (p = 0.049) in EA families (Supplementary Table S9).

### Rare variants in established genes from GWAS

We interrogated our individual variant and gene-based aggregate association tests for 299 previously associated AD genes. Among the SNVs and indels, a total of 1,172 variants with MAF < 0.05 and annotated as either HIGH or MODERATE impact are located within 253 interrogated AD genes (Supplementary Table S10). Five of these variants were at least suggestively significant (p<8.9 × 10^−4^) in single variant testing. The most significant associations included the *TREM2* R47H missense mutation (p = 4.8 × 10^−12^) and *ABCA7* frameshift mutation E709fs (p = 4.3 × 10^−6^) which was previously associated with AD in Belgian families [40]. Additional notable signals included variants in *SORL1* A528T (p = 8.7 × 10^−5^), which was previously associated with AD in a CH population [15], and *ACP2* D353E (p = 7.8 × 10^−4^). Perturbation of murine Acp2 causes lysosomal storage deficits, kyphoscoliosis, cerebellar abnormalities, and ataxia [44, 45].

For gene-based tests, we aggregated variants on the basis of annotated function, and examined only genes with more than one contributing variant and a cMAC ≥ 10 Of the 299 AD genes, tests were performed on 281 genes aggregating high or moderate impact variants and 86 genes limited to high impact variants. Among these, 13 unique genes surpassed suggestive significance thresholds for high (p < 1.16 × 10^−2^) or high-moderate (p = 3.56 × 10^−3^) impact variants. The strongest associations were observed for moderate impact variants in *TREM2* (p = 4.81 × 10^−12^) and *SORL1* (p = 8.68 × 10^−5^), and a high impact variant in *ABCA7* (p = 4.33 × 10^−6^). Other noteworthy signals included moderate impact variants in *NUP88* (p = 4.63 × 10^−4^) and *ACP2* (p = 7.80 × 10^−4^).

## Discussion

Our WES study, the largest for AD conducted to date, identified novel associations with variants in three genes not previously implicated in AD including one common nearly exome-wide significant variant each in *IGHG3* (p = 9.8 × 10^−7^) and *STAG3* (p = 8.8 × 10^−7^), and one rare exome-wide significant variant in *AC099552.4* (p = 1.2 × 10^−7^). We also observed a gene-wide significant association with *ZNF655* in a gene-based test including nine high-impact rare variants (p = 5.0 × 10^−6^). These results remained significant after multiple test correction and were confirmed in or strengthened by a replication sample comprised of four independent datasets, with the exception of the variant in *AC099552.4* which was invariant in the replication samples. We also confirmed associations with common and rare variants in several previously established AD genes including *ABCA7*, *APOE*, *HLA-DPA1, MS4A6A, PILRA*, *SORL1* and *TREM2*.

*ZNF655* is expressed in brain and encodes the Vav-interacting Krüppel-like factor 1 [46]. Krüppel-like factors (KLFs) are zinc finger-containing transcription factors that regulate diverse biological processes, including proliferation, differentiation, growth, development, survival, and responses to external stress [47]. Several KLFs have been shown to participate in neuronal morphogenesis and to control the regenerative capacity of neurons in the central nervous system. *AC099552.4* is a long non-coding RNA, an abundant class of RNA sequences which regulate gene transcription and expression [48] and impact neuronal development, neuroplasticity, and cognition [49]. Non-coding RNA-dependent regulation affecting AD-related processes has been demonstrated for *SORL1* [50] and in a triple transgenic model of AD [51].

*IGHG3* encodes immunoglobulin heavy constant gamma 3 and is a member of the IgG family for which antibodies have been shown to cross-react with fibril and oligomer amyloid-β aggregates [52] leading to speculation that Immunoglobulin GM (γ marker) genes contain functional risk and protective factors for AD [53]. The anti-amyloidogenic activity of IgG appears to be an inherent property of free human IgG heavy chains [54]. Recent analysis of structural variants in whole genome sequence data for 578 members of 101 families with multiple AD subjects included in the ADSP [26] yielded additional evidence supporting *IGHG3* as an AD risk locus. A total of nine distinct deletions in the *IGH* region were identified as disproportionately represented in AD cases compared to controls. One of these is a 188 bp deletion that was observed in 35 AD cases and 8 controls and is located 592 bp from the AD-associated SNV (rs12890621) in this study. This deletion eliminates a large portion of *IGHG3* intron 2 and exon 3 (reference transcript ENST00000390551), and is predicted to have high impact on the encoded product. It is unlikely that the deletion and rs12890621 tag the same effector of AD risk because the deletion is rare, whereas rs12890621 is more common (MAF = 0.0475 in EA subjects according to the ExAC database). Of note, a nearby pseudogene in the IgG family, *IGHV1–67*, located approximately 350 kb from *IGHG3*, has been previously reported in a gene-wide association study conducted by the International Genetics of Alzheimer’s Project (IGAP) [1].

The association with the common synonymous variant in *STAG3* (rs1043915, MAF = 0.26) is not independent of the finding with a common SNV in *PILRA*, a previously reported AD-associated gene [43] located in an established AD locus [9]. However, rare variants in *STAG3* identified by WGS showed evidence of co-segregation with AD in CH families suggesting the possibility that *STAG3* has a distinct mechanistic role in AD. *STAG3*, stromal antigen 3, encodes a subunit of the cohesin complex which regulates the cohesion of sister chromatids during cell division. Whether the association with AD observed here is mediated at least in part through *STAG3* function or simply reflects linkage disequilibrium with other causal variants/genes in the region remains to be established. Rare coding *STAG3* variants have been identified in primary ovarian insufficiency [55]. Although *STAG3* is expressed in the brain, its role remains unclear. Interestingly, data from GTEx show that the associated variant is an eQTL for multiple genes in various brain tissues, including *STAG3*, *AGFG2, GAL3ST4, GATS*, and *PVRIG*. In a mouse model of diabetes, micro-vascular damage in the neurovascular unit of the retina was associated with alteration in *STAG3* expression [56].

A variant in *NSF* showed nominally significant evidence of association in the replication sample (p = 0.014) in the model adjusting for age, sex, and *APOE* ε4 status, whereas the result in the discovery sample was observed in the model without these covariates. *NSF* encodes N-ethylmaleimide sensitive factor, vesicle fusing ATPase is involved in membrane trafficking of proteins and neuro-transmitter release [57], and has been observed in brain homogenates of cases of familial neuronal intranuclear inclusion disease [58]. NSF SNVs have been associated with cocaine dependence [59] and its expression is reduced in prefrontal cortex in schizophrenia patients [60]. Vesicular trafficking has an important role in AD exemplified by genetic and biological evidence for neuronal sorting proteins including *SORL1* [61–63].

We were unable to replicate variants at five loci that showed significant association in the discovery sample (p ≤ 5.0 × 10^−6^). Failure to replicate findings for the *OPRL1* and *DTNBP1* variants may be due to their lower MAF and, hence, uncertainty in the imputation quality and lack of imputed indels in the ADGC GWAS replication sample. Nonetheless, both of these genes are potentially attractive biological candidates. Opioid related nociceptin receptor 1 modulates a variety of biological functions and neurobehavior, including learning and memory, and inflammatory and immune functions [64, 65]. *DTNBP1* encodes the dystrobrevin binding protein 1 which has been genetically linked to multiple psychiatric disorders, as well as cognitive and memory functions in healthy human subjects [66, 67].

Analysis of rare variants in the regions of genes previously identified as related to AD by GWAS revealed genome-wide significant or suggestive evidence of association in established genes including *TREM2*, *SORL1*, and *ABCA7*. In addition, notable associations were observed with other genes in these regions not previously linked to AD including *TREML4, SPPL2A,* and *AP4M1* (Supplementary Table S10). *TREML4* is located near *TREM2* and encodes a *TREM* family receptor that, similar to *TREM2*, is expressed on the surface of myeloid cells and participates in the phagocytic clearance of dead cells [68]. *SPPL2A* encodes an endosomal-lysosomal protease and presenilin homolog that regulates B-cell homeostasis in vivo [69]. Homozygous mutations in *AP4M1*, located in the region including *PILRA* and *STAG3*, cause spastic tetraplegia, intellectual disability, and white matter loss [70]. Its encoded protein is a component of the AP-4 trafficking complex that regulates APP processing and beta-amyloid secretion in cell models [71]. Further studies are needed to conclude whether the association findings in this latter group of genes are robust and warrant experiments to determine their functional relevance to AD.

Notably, there is little overlap of our results with findings of large GWAS focused on common variants [1, 2, 9]. This is due in part to our focus on only infrequent or rare variants (MAF < 0.05) that are functionally-annotated to be of at least moderate impact and may not have been well covered by GWAS arrays or imputation. With the notable exception of *APOE*, common variants associated with AD have very modest effect on risk (OR < 1.3) [9], and all but a few of these associations [4, 5, 8, 10, 12] required a sample between two and nearly seven times larger than the sample in this study to have sufficient power to detect them [1, 2, 9].

Our study has several notable strengths and limitations. The ascertainment scheme for this sample is optimal for detection of association with both risk and protective variants for AD [26]. Specifically, the AD cases were selected to have relatively early onset (with a minimum age of 65) and a lower frequency of the *APOE* ε4 allele with the expectation that they were likely to be more enriched for rare high-penetrant AD risk-variants compared to most late-onset AD cases. Controls were selected to be as old as possible with preference given to those having at least one *APOE* ε4 allele to enrich this group for protective variants. However, this scheme introduced confounding between age and AD status which reduced power for detecting associations. To overcome this limitation, we included a model without age adjustment which yielded the largest number of new association findings including several that were replicated in independent datasets which were analyzed with age adjustment. Thus, it was important to include models which did or did not include a covariate for age in order to account for confounding with AD status as well as age-dependent effects of the genetic factor. Despite simulations showing that this sample had sufficient power to detect associations with variants whose frequencies were as low as 0.005 and an effect size greater than 1.8 [26], the number of novel rare variant findings were few. We also acknowledge that p-value thresholds did not account for the number of models tested, however the models are highly correlated (Supplementary Table S2).

The inclusion of CH participants who were a pivotal portion of a multi-ethnic sample leading to the discovery of common variant associations in other AD loci, most notably *SORL1* [62], but for rare variant discovery these samples may have reduced power by increasing genetic heterogeneity of the total sample. This conclusion is consistent with observations of few novel findings in this WES study showing discernable contributions by the CH dataset and by discovery of novel rare variant associations in a whole genome sequence study that were unique to EA and CH families, respectively [34, 35]. Nonetheless, the non-Hispanic portion of our sample was sufficiently large to detect multiple novel associations. Our findings suggest that additional large and ancestrally diverse cohorts with deep sequence data will need to be examined for replication and to provide a larger discovery sample.

Successful replication of only some of the most significant findings in novel genes not in the *APOE* region (4/8 individual variants in Table 2, 1/18 genes in Table 3) is somewhat concerning but highlights the difficulty of designing well-powered replication studies of sequencing findings. Although it is possible that some of these findings are false positives, we acknowledge that the size of the WES replication samples combined (2,778 AD cases, 7,262 controls) was inadequate. In addition, many rare variants were not well-imputed or, in the case of most indels, not imputed at all in the ADGC GWAS dataset, despite the use of the HRC reference panel which contains haplotypes derived from whole genome sequence data for more than 30,000 individuals who were not ascertained for AD research. Thus, additional large WES samples will need to be studied to obtain definitive evidence about findings that did not replicate.

In summary, our significant association findings with functional rare variants in novel genes provide further support for the roles of neuroinflammation (*IGHG3*) and transcriptional regulation (*AC099552.4* and *ZNF655*) in AD. In addition, we identified many novel associations with rare functional variants in previously established AD genes. In most cases, these rare variants do not explain association signals that were previously identified by GWAS with common and predominantly non-functional variants. Hence, many of our findings will provide insight into disease mechanisms and targets for biological experiments to gain further understanding about the role of these genes in AD pathogenesis. However, other deep sequencing approaches (e.g., whole genome, target gene resequencing) will be needed to identify variants which account for association signals in non-coding regions and the contribution of structural variants (e.g., larger insertions and deletions, copy number variants, etc.) to AD risk.

## Supplementary Material

Supp. Material

Supp. Tables

## Figures and Tables

**Table 1:** Participant Characteristics

A. Discovery Sample									
		AD Cases (N = 5,740)				Cognitively Normal Controls (N = 5,096)			
Ancestry	Sampling	N	Age (mean)	Sex (%F)	APOE E4 (%carrier)	N	Age. (mean)	Sex (%F)	APOE E4 (%carrier)
EA	Case-Control	5,015	75.25	55.8%	40.6%	4,919	86.53	59.2%	14.4%
EA	Enriched	507	83.61	63.3%	66.9%	NA	NA	NA	NA
Hispanic	Case-Control	46	72.59	71.7%	43.5%	6	85.94	66.7%	16.7%
Hispanic	Enriched	172	75.45	61.6%	39.5%	171	73.46	60.8%	39.2%

B. Replication Sample									
		AD Cases (N = 12,121)				Cognitively Normal Controls (N = 18,789)			
		N	Age (mean)	Sex (% F)	APOE ε4 (% carrier)	N	Age (mean)	Sex (% F)	APOE ε4 (% carrier)
CHARGE	WES	612	81	67%	54%	1,836	80	58%	24%
ADES-FR	WES	1,142	74	64%	49%	1,104	80	58%	22%
FinnAD	WES	1,024	74	62%	--	4,322	71	51%	--
ADGC	GWAS	9,343	74	54%	64%	11,527	75	54%	25%

**Table 2:** Associations with Individual Variants outside the *APOE* region

		ADSP Discovery Meta			All Replication (ADGC+CHARGE.+ADES-FR+FinnAD)			Discovery + All Replication		
Name	gene	MAC (EA/CH)	best P	Model (group)	MAC	P Model 1	P Model 2	P Model 0	P Model 1	P Model 2
6:41129252:C:T (R47H)	TREM2	120/0	**4.8E-12**	0 (EA)	224	**1.6E-06**	**2.7E-06**	**3.2E-16**	**2.8E-10**	**1.6E-10**
7:154988675:G:A	AC099552.4	10/0	**1.2E-07**	2 (EA)	0	NA	NA	*1.3E-02*	***2.0E-07***	***1.2E-07***
7:99971313:T:C (rs2405442)	PILRA	6,219/219	**1.7E-07**	0 (EA)	22,798	5.3E-05	2.3E-05	**9.5E-10**	1.1E-06	5.0E-07
20:62729814:C:T (rs148484121)	OPRL1	61/4	5.8E-07	1 (all)	111	3.4E-01	5.6E-01	3.7E-03	1.4E-04	4.5E-04
11:59940599:T:A (rs7232)	MS4A6A	7,540/258	7.7E-07	0 (all)	20,963	**1.4E-11**	**3.1E-09**	**5.6E-17**	**3.8E-14**	**2.6E-11**
17:44828931:G:A (rs199533)	NSF	4,238/135	1.3E-06	0 (all)	11,120	2.5E-01	1.4E-02	2.1E-04	1.6E-02	1.9E-04
14:106235767:C:T (rs77307099)	IGHG3	6,200/176	1.9E-06	0 (all)	721	4.0E-01	3.5E-01	**1.4E-06**	1.3E-04	7.9E-05
14:106235766:G:A (rs78376194)	IGHG3	6,202/176	1.9E-06	0 (all)	719	4.2E-01	3.6E-01	**1.5E-06**	1.4E-04	8.5E-05
6:15638035:C:T (rs77460377)	DTNBP1	16/3	1.9E-06	2 (all)	35	8.5E-01	8.7E-01	8.7E-02	5.2E-03	3.0E-03
6:33041297:G:A (rs112178281)	HLA-DPA1	10/0	2.9E-06	1 (EA)	6	7.5E-01	9.2E-01	1.4E-01	2.1E-05	2.0E-05
11:59945745:T:C (rs12453)	MS4A6A	8,265/258	3.2E-06	0 (EA)	23,420	**4.7E-11**	**3.0E-08**	**1.4E-15**	**6.0E-13**	**1.2E-09**
3:195506101:T:A	MUC4	38/6	3.8E-06	1 (all)	0	NA	NA	*3.0E-04*	*3.8E-06*	*8.1E-06*
10:88446985:T:C (rs76615432)	LDB3	760/62	5.0E-06	1 (CH)	2,303	5.3E-01	5.9E-01	7.0E-01	6.3E-01	6.4E-01
19:1047507:AGGAGCAG:A	ABCA7	67/0	4.3E-06	0 (EA)	11	8.8E-02	9.7E-02	2.4E-04	1.6E-02	1.7E-02
14:106236128:T:A (rs12890612)	IGHG3	6,395/369	4.5E-06	0 (all)	1,473	8.5E-02	7.5E-02	**9.8E-07**	**8.0E-05**	6.4E-05
7:99799845:T:A (rs104395)	STAG3	5,248/248	5.5E-06	0 (EA)	15,948	3.0E-03	1.2E-03	**8.8E-07**	1.2E-04	4.0E-05

Table shows variants with *P* < 6.1 × 10^−6^ in EA, CH^,^ or combined strata in the discovery sample. Exome-wide significant results (*P* < 3.1 × 10^−7^) and suggestive results which improved in meta analysis of discovery + replication data are highlighted in bold. Results without variation data in the replication datasets are indicated in *italics*

**Table 3:** Gene-based Association Results

		ADSP Discovery Meta			All Replication			Discovery + All Replication			
Gene	Variants b	SNVs	best P	Model	SNVs	P Model 1	P Model 2	SNVs	P Model 0	P Model 1	P Model 2
TREM2	High-Mod	50	**1.8E-11**	0	33	**9.3E-10**	**5.4E-09**	65	**2.0E-17**	**3.8E-11**	**6.0E-11**
CBLC ^a^	High-Mod	44	**1.1E-07**	0	35	**2.5E-20**	**6.7E-03**	61	**1.0E-27**	**6.1E-22**	4.9E-02
OPRL1	High-Mod	42	**2.6E-06**	1	37	1.3E-01	3.0E-01	64	8.3E-03	5.4E-04	1.7E-03
CBX3	High-Mod	8	6.0E-05	0	10	1.3E-01	2.8E-01	17	4.9E-04	4.6E-02	6.1E-02
BCAM ^a^	High-Mod	90	5.2E-04	1	88	**4.7E-19**	3.7E-03	144	**3.5E-27**	**2.8E-20**	4.8E-02
GAS2L2	High	7	**3.9E-06**	2	5	5.1E-02	6.7E-02	10	4.5E-01	3.9E-02	2.9E-02
ZNF655	High	9	2.8E-05	0	6	3.2E-02	3.4E-02	13	**7.9E-06**	8.4E-04	3.4E-04
RHBDD1	High	2	3.2E-05	2	4	8.8E-01	9.8E-01	5	3.5E-01	4.8E-01	2.7E-01
SIRPB1	High	6	8.0E-05	2	3	9.2E-01	7.9E-01	8	6.4E-01	3.0E-01	2.6E-01
RPS16	High	5	1.6E-04	2	2	7.4E-01	4.2E-01	5	4.4E-02	7.7E-03	6.5E-03
ABCA7	LoF	43	**2.1E-06**	0	16	1.5E-01	1.1E-01	51	1.2E-04	1.2E-03	3.4E-04
GAS2L2	LoF	7	**3.9E-06**	2	3	3.9E-02	4.8E-02	8	5.2E-01	4.3E-02	2.5E-02
ZNF655	LoF	8	1.9E-05	0	4	3.9E-02	3.0E-02	10	**5.0E-06**	4.6E-04	2.0E-04
RPS16	LoF	3	1.6E-04	2	2	7.4E-01	4.2E-01	3	4.1E-02	7.9E-03	6.4E-03

Table shows genes with *P*-value < 5.7 × 10^−5^ (High-Mod^)^, 2.2 × 10^−4^ (High), or 2.8 × 10^−4^ (LoF) in the total discovery sample. Results surpassing discovery stage Bonferroni corrected significance thresholds -- *P* = 2.8 × 10^−6^ (High-Mod^),^ 1.1 × 10^−5^ (High), and 1.4 × 10^−5^ (LoF) – are indicated in bold.

a located in *APOE* region

b type of functional variants included in gene-based test

## References

[1] Escott-Price V, Bellenguez C, Wang LS, Choi SH, Harold D, Jones L, Gene-wide analysis detects two new susceptibility genes for Alzheimer’s disease. PLoS One 2014;9:e94661. 24922517 10.1371/journal.pone.0094661 PMC4055488

[2] Escott-Price V, Sims R, Bannister C, Harold D, Vronskaya M, Majounie E, Common polygenic variation enhances risk prediction for Alzheimer’s disease. Brain 2015;138:3673–84. 26490334 10.1093/brain/awv268 PMC5006219

[3] Guerreiro R, Wojtas A, Bras J, Carrasquillo M, Rogaeva E, Majounie E, TREM2 variants in Alzheimer’s disease. N Engl J Med 2013;368:117–27. 23150934 10.1056/NEJMoa1211851 PMC3631573

[4] Harold D, Abraham R, Hollingworth P, Sims R, Gerrish A, Hamshere ML, Genome-wide association study identifies variants at CLU and PICALM associated with Alzheimer’s disease. Nat Genet 2009;41:1088–93. 19734902 10.1038/ng.440 PMC2845877

[5] Hollingworth P, Harold D, Sims R, Gerrish A, Lambert JC, Carrasquillo MM, Common variants at ABCA7, MS4A6A/ MS4A4E, EPHA1, CD33 and CD2AP are associated with Alzheimer’s disease. Nat Genet 2011;43:429–35. 21460840 10.1038/ng.803 PMC3084173

[6] Jonsson T, Atwal JK, Steinberg S, Snaedal J, Jonsson PV, Bjornsson S, A mutation in APP protects against Alzheimer’s disease and age-related cognitive decline. Nature 2012;488:96–99. 22801501 10.1038/nature11283

[7] Jonsson T, Stefansson H, Steinberg S, Jonsdottir I, Jonsson PV, Snaedal J, Variant of TREM2 associated with the risk of Alzheimer’s disease. N Engl J Med 2013;368:107–16. 23150908 10.1056/NEJMoa1211103 PMC3677583

[8] Lambert JC, Heath S, Even G, Campion D, Sleegers K, Hiltunen M, Genome-wide association study identifies variants at CLU and CR1 associated with Alzheimer’s disease. Nat Genet 2009;41:1094–9. 19734903 10.1038/ng.439

[9] Lambert JC, Ibrahim-Verbaas CA, Harold D, Naj AC, Sims R, Bellenguez C, Meta-analysis of 74,046 individuals identifies 11 new susceptibility loci for Alzheimer’s disease. Nat Genet 2013;45:1452–8. 24162737 10.1038/ng.2802 PMC3896259

[10] Naj AC, Jun G, Beecham GW, Wang LS, Vardarajan BN, Buros J, Common variants at MS4A4/MS4A6E, CD2AP, CD33 and EPHA1 are associated with late-onset Alzheimer’s disease. Nat Genet 2011;43:436–41. 21460841 10.1038/ng.801 PMC3090745

[11] Ruiz A, Heilmann S, Becker T, Hernandez I, Wagner H, Thelen M, Follow-up of loci from the International Genomics of Alzheimer’s Disease Project identifies TRIP4 as a novel susceptibility gene. Transl Psychiatry 2014;4:e358. 24495969 10.1038/tp.2014.2 PMC3944635

[12] Seshadri S, Fitzpatrick AL, Ikram MA, DeStefano AL, Gudnason V, Boada M, Genome-wide analysis of genetic loci associated with Alzheimer disease. JAMA 2010;303:1832–40. 20460622 10.1001/jama.2010.574 PMC2989531

[13] Gatz M, Reynolds CA, Fratiglioni L, Johansson B, Mortimer JA, Berg S, Role of genes and environments for explaining Alzheimer disease. Arch Gen Psychiatry 2006;63:168–74. 16461860 10.1001/archpsyc.63.2.168

[14] Vardarajan BN, Ghani M, Kahn A, Sheikh S, Sato C, Barral S, Rare coding mutations identified by sequencing of Alzheimer disease genome-wide association studies loci. Ann Neurol 2015;78:487–98. 26101835 10.1002/ana.24466 PMC4546546

[15] Vardarajan BN, Zhang Y, Lee JH, Cheng R, Bohm C, Ghani M, Coding mutations in SORL1 and Alzheimer disease. Ann Neurol 2015;77:215–27. 25382023 10.1002/ana.24305 PMC4367199

[16] Steinberg S, Stefansson H, Jonsson T, Johannsdottir H, Ingason A, Helgason H, Loss-of-function variants in ABCA7 confer risk of Alzheimer’s disease. Nat Genet 2015;47:445–7. 25807283 10.1038/ng.3246

[17] Logue MW, Schu M, Vardarajan BN, Farrell J, Bennett DA, Buxbaum JD, Two rare AKAP9 variants are associated with Alzheimer’s disease in African Americans. Alzheimers Dement 2014;10:609–18. 25172201 10.1016/j.jalz.2014.06.010 PMC4253055

[18] Jun G, Asai H, Zeldich E, Drapeau E, Chen C, Chung J, PLXNA4 is associated with Alzheimer disease and modulates tau phosphorylation. Ann Neurol 2014;76:379–92. 25043464 10.1002/ana.24219 PMC4830273

[19] Wetzel-Smith MK, Hunkapiller J, Bhangale TR, Srinivasan K, Maloney JA, Atwal JK, A rare mutation in UNC5C pre-disposes to late-onset Alzheimer’s disease and increases neuronal cell death. Nat Med 2014;20:1452–7. 25419706 10.1038/nm.3736 PMC4301587

[20] Bodmer W, Bonilla C. Common and rare variants in multifactorial susceptibility to common diseases. Nat Genet 2008;40:695–701. 18509313 10.1038/ng.f.136 PMC2527050

[21] Schork NJ, Murray SS, Frazer KA, Topol EJ. Common vs. rare allele hypotheses for complex diseases. Curr Opin Genet Dev 2009;19:212–9. 19481926 10.1016/j.gde.2009.04.010 PMC2914559

[22] Surakka I, Horikoshi M, Magi R, Sarin AP, Mahajan A, Lagou V, The impact of low-frequency and rare variants on lipid levels. Nat Genet 2015;47:589–97. 25961943 10.1038/ng.3300 PMC4757735

[23] Pritchard JK. Are rare variants responsible for susceptibility to complex diseases? Am J Hum Genet 2001;69:124–37. 11404818 10.1086/321272 PMC1226027

[24] Rabbani B, Tekin M, Mahdieh N. The promise of whole-exome sequencing in medical genetics. J Hum Genet 2014;59: 5–15. 24196381 10.1038/jhg.2013.114

[25] McCarthy S, Das S, Kretzschmar W, Delaneau O, Wood AR, Teumer A, A reference panel of 64,976 haplotypes for genotype imputation. Nat Genet 2016;48:1279–83. 27548312 10.1038/ng.3643 PMC5388176

[26] Beecham GW, Bis JC, Martin ER, Choi S-H, DeStefano A, van Duijn C, The Alzheimer’s Disease Sequencing Project: study design and sample selection. Neurol Genet 2017;3:e194. 29184913 10.1212/NXG.0000000000000194 PMC5646177

[27] Mirra SS, Hart MN, Terry RD. Making the diagnosis of Alzheimer’s disease. A primer for practicing pathologists. Arch Pathol Lab Med 1993;117:132–44. 8427562

[28] Braak H, Braak E. Neuropathological stageing of Alzheimer-related changes. Acta Neuropathol 1991;82:239–59. 1759558 10.1007/BF00308809

[29] umley T, Brody J, Dupuis J, Cupples A Meta-analysis of a rare-variant association test: University of Auckland; 2012 http://stattech.wordpress.fos.auckland.ac.nz/files/2012/11/skat-meta-paper.pdf. Technical report.

[30] Lee S, Emond MJ, Bamshad MJ, Barnes KC, Rieder MJ, Nickerson DA, Optimal unified approach for rare-variant association testing with application to small-sample case-control whole-exome sequencing studies. Am J Hum Genet 2012;91:224–37. 22863193 10.1016/j.ajhg.2012.06.007 PMC3415556

[31] Bellenguez C, Charbonnier C, Grenier-Boley B, Quenez O, Le Guennec K, Nicolas G, Contribution to Alzheimer’s disease risk of rare variants in TREM2, SORL1, and ABCA7 in 1779 cases and 1273 controls. Neurobiol Aging 2017;59:220 e221–220 e229. 10.1016/j.neurobiolaging.2017.07.001 28789839

[32] Loh PR, Danecek P, Palamara PF, Fuchsberger C, A Reshef Y, K Finucane H, Reference-based phasing using the Haplotype Reference Consortium panel. Nat Genet 2016;48: 1443–8. 27694958 10.1038/ng.3679 PMC5096458

[33] Skol AD, Scott LJ, Abecasis GR, Boehnke M. Joint analysis is more efficient than replication-based analysis for two-stage genome-wide association studies. Nat Genet 2006;38:209–13. 16415888 10.1038/ng1706

[34] Blue EE, Bis JC, Dorschner MO, Tsuang D, Barral SM, Beecham G, Genetic variation in genes underlying diverse dementias may explain a small proportion of cases in the Alzheimer’s Disease Sequencing Project. Dement Ger Cog Disorders 2018;45: 1–17. 45 10.1159/000485503 PMC5971141 29486463

[35] Beecham GW, Vardarajan BN, Blue E, Barral S, Haines JL, Bush WS, Whole-genome sequencing in familial late-onset Alzheimer’s disease identifies variation in AD candidate genes. Alzheimer Dement 2017;13:P571–P572.

[36] Yan Q, Tiwari HK, Yi N, Gao G, Zhang K, Lin WY, A sequence kernel association test for dichotomous traits in family samples under a generalized linear mixed model. Hum Hered 2015;79:60–68. 25791389 10.1159/000375409 PMC4825859

[37] Liu JZ, Erlich Y, Pickrell JK. Case-control association mapping by proxy using family history of disease. Nat Genet 2017;49: 325–31. 28092683 10.1038/ng.3766

[38] Neph S, Kuehn MS, Reynolds AP, Haugen E, Thurman RE, Johnson AK, BEDOPS: high-performance genomic feature operations. Bioinformatics 2012;28:1919–20. 22576172 10.1093/bioinformatics/bts277 PMC3389768

[39] Quinlan AR, Hall IM. BEDTools: a flexible suite of utilities for comparing genomic features. Bioinformatics 2010;26:841–2. 20110278 10.1093/bioinformatics/btq033 PMC2832824

[40] Harrow J, Frankish A, Gonzalez JM, Tapanari E, Diekhans M, Kokocinski F, GENCODE: the reference human genome annotation for The ENCODE Project. Genome Res 2012;22: 1760–74. 22955987 10.1101/gr.135350.111 PMC3431492

[41] Pruim RJ, Welch RP, Sanna S, Teslovich TM, Chines PS, Gliedt TP, LocusZoom: regional visualization of genome-wide association scan results. Bioinformatics 2010;26:2336–7. 20634204 10.1093/bioinformatics/btq419 PMC2935401

[42] Cuyvers E, De Roeck A, Van den Bossche T, Van Cauwenberghe C, Bettens K, Vermeulen S, Mutations in ABCA7 in a Belgian cohort of Alzheimer’s disease patients: a targeted resequencing study. Lancet Neurol 2015;14:814–22. 26141617 10.1016/S1474-4422(15)00133-7

[43] Logue MW, Schu M, Vardarajan BN, Farrell J, Lunetta KL, Jun G, A search for genetic risk variants for age-related macular degeneration in Alzheimer disease genes and pathways. Neurobiol Aging 2014;35:1510 e7–e1510.e18. 10.1016/j.neurobiolaging.2013.12.007 PMC3961547 24439028

[44] Saftig P, Hartmann D, Lullmann-Rauch R, Wolff J, Evers M, Koster A, Mice deficient in lysosomal acid phosphatase develop lysosomal storage in the kidney and central nervous system. J Biol Chem 1997;272:18628–35. 9228031 10.1074/jbc.272.30.18628

[45] Mannan AU, Roussa E, Kraus C, Rickmann M, Maenner J, Nayernia K, Mutation in the gene encoding lysosomal acid phosphatase (Acp2) causes cerebellum and skin malformation in mouse. Neurogenetics 2004;5:229–38. 15503243 10.1007/s10048-004-0197-9

[46] Houlard M, Romero-Portillo F, Germani A, Depaux A, Regnier-Ricard F, Gisselbrecht S, Characterization of VIK-1: a new Vav-interacting Kruppel-like protein. Oncogene 2005;24:28–38. 15558030 10.1038/sj.onc.1208043

[47] McConnell BB, Yang VW. Mammalian Kruppel-like factors in health and diseases. Physiol Rev 2010;90:1337–81. 20959618 10.1152/physrev.00058.2009 PMC2975554

[48] Goodrich JA, Kugel JF. Non-coding-RNA regulators of RNA polymerase II transcription. Nature Rev Molec Cell Biol 2006;7: 612–6. 16723972 10.1038/nrm1946

[49] Butler AA, Webb WM, Lubin FD. Regulatory RNAs and control of epigenetic mechanisms: expectations for cognition and cognitive dysfunction. Epigenomics 2016;8:135–51. 26366811 10.2217/epi.15.79 PMC5514977

[50] Ciarlo E, Massone S, Penna I, Nizzari M, Gigoni A, Dieci G, An intronic ncRNA-dependent regulation of SORL1 expression affecting Aβ formation is upregulated in post-mortem Alzheimer’s disease brain samples. Dis Model Mech 2013;6:424–33. 22996644 10.1242/dmm.009761 PMC3597024

[51] Lee DY, Moon J, Lee ST, Jung KH, Park DK, Yoo JS, Distinct expression of long non-coding RNAs in an Alzheimer’s disease model. J Alzheimers Dis 2015;45:837–49. 25624420 10.3233/JAD-142919

[52] O’Nuallain B, Acero L, Williams AD, Koeppen HP, Weber A, Schwarz HP, Human plasma contains cross-reactive Abeta conformer-specific IgG antibodies. Biochemistry 2008;47: 12254–6. 18956886 10.1021/bi801767k

[53] Pandey JP. Immunoglobulin GM genes as functional risk and protective factors for the development of Alzheimer’s disease. J Alzheimers Dis 2009;17:753–6. 19542624 10.3233/JAD-2009-1094

[54] Adekar SP, Klyubin I, Macy S, Rowan MJ, Solomon A, Dessain SK, Inherent anti-amyloidogenic activity of human immunoglobulin gamma heavy chains. J Biol Chem 2010;285: 1066–74. 19889627 10.1074/jbc.M109.044321 PMC2801233

[55] He WB, Banerjee S, Meng LL, Du J, Gong F, Huang H, Whole-exome sequencing identifies a homozygous donor splice-site mutation in STAG3 that causes primary ovarian insufficiency. Clin Genet 2018;93-340–4. 28393351 10.1111/cge.13034

[56] Friedrichs P, Schlotterer A, Sticht C, Kolibabka M, Wohlfart P, Dietrich A, Hyperglycaemic memory affects the neurovascular unit of the retina in a diabetic mouse model. Diabetologia 2017;60:1354–8. 28321468 10.1007/s00125-017-4254-y

[57] Pittaluga A, Feligioni M, Longordo F, Luccini E, Raiteri M. Trafficking of presynaptic AMPA receptors mediating neuro-transmitter release: neuronal selectivity and relationships with sensitivity to cyclothiazide. Neuropharmacology 2006;50:286–96. 16242162 10.1016/j.neuropharm.2005.09.004

[58] Pountney DL, Raftery MJ, Chegini F, Blumbergs PC, Gai WP. NSF, Unc-18–1, dynamin-1 and HSP90 are inclusion body components in neuronal intranuclear inclusion disease identified by anti-SUMO-1-immunocapture. Acta Neuropathol 2008;116: 603–14. 18836734 10.1007/s00401-008-0437-4

[59] Fernandez-Castillo N, Cormand B, Roncero C, Sanchez-Mora C, Grau-Lopez L, Gonzalvo B, Candidate pathway association study in cocaine dependence: the control of neurotransmitter release. World J Biol Psychiatry 2012;13:126–34. 21426264 10.3109/15622975.2010.551406

[60] Imai C, Sugai T, Iritani S, Niizato K, Nakamura R, Makifuchi T, A quantitative study on the expression of synapsin II and N-ethylmaleimide-sensitive fusion protein in schizophrenic patients. Neurosci Lett 2001;305:185–8. 11403936 10.1016/s0304-3940(01)01844-4

[61] Reitz C, Cheng R, Rogaeva E, Lee JH, Tokuhiro S, Zou F, Meta-analysis of the association between variants in SORL1 and Alzheimer disease. Arch Neurol 2011;68:99–106. 21220680 10.1001/archneurol.2010.346 PMC3086666

[62] Rogaeva E, Meng Y, Lee JH, Gu Y, Kawarai T, Zou F, The neuronal sortilin-related receptor SORL1 is genetically associated with Alzheimer disease. Nat Genet 2007;39:168–77. 17220890 10.1038/ng1943 PMC2657343

[63] Willnow TE, Andersen OM. Sorting receptor SORLA--a trafficking path to avoid Alzheimer disease. J Cell Sci 2013;126: 2751–60. 23813966 10.1242/jcs.125393

[64] Gavioli EC, de Medeiros IU, Monteiro MC, Calo G, Romao PR. Nociceptin/orphanin FQ-NOP receptor system in inflammatory and immune-mediated diseases. Vitam Horm 2015;97:241–66. 25677775 10.1016/bs.vh.2014.11.003

[65] Abdel-Mouttalib O Nociceptin/orphanin-FQ modulation of learning and memory. Vitam Horm 2015;97:323–45. 25677778 10.1016/bs.vh.2014.10.006

[66] Hashimoto R, Noguchi H, Hori H, Nakabayashi T, Suzuki T, Iwata N, A genetic variation in the dysbindin gene (DTNBP1) is associated with memory performance in healthy controls. World J Biol Psychiatry 2010;11:431–8. 19353385 10.1080/15622970902736503

[67] Hashimoto R, Noguchi H, Hori H, Ohi K, Yasuda Y, Takeda M, Association between the dysbindin gene (DTNBP1) and cognitive functions in Japanese subjects. Psychiatry Clin Neurosci 2009;63:550–6. 19496996 10.1111/j.1440-1819.2009.01985.x

[68] Hemmi H, Idoyaga J, Suda K, Suda N, Kennedy K, Noda M, A new triggering receptor expressed on myeloid cells (Trem) family member, Trem-like 4, binds to dead cells and is a DNAX activation protein 12-linked marker for subsets of mouse macro-phages and dendritic cells. J Immunol 2009;182:1278–86. 19155473 10.4049/jimmunol.182.3.1278 PMC2843158

[69] Mentrup T, Fluhrer R, Schroder B. Latest emerging functions of SPP/SPPL intramembrane proteases. Eur J Cell Biol 2017;96: 372–82. 28366434 10.1016/j.ejcb.2017.03.002

[70] Verkerk AJ, Schot R, Dumee B, Schellekens K, Swagemakers S, Bertoli-Avella AM, Mutation in the AP4M1 gene provides a model for neuroaxonal injury in cerebral palsy. Am J Hum Genet 2009;85:40–52. 19559397 10.1016/j.ajhg.2009.06.004 PMC2706965

[71] Toh WH, Tan JZ, Zulkefli KL, Houghton FJ, Gleeson PA. Amyloid precursor protein traffics from the Golgi directly to early endosomes in an Arl5b- and AP4-dependent pathway. Traffic 2017;18:159–75. 28000370 10.1111/tra.12465

