# Correction: Whole exome sequencing study identifies novel rare and common Alzheimer’s-Associated variants involved in immune response and transcriptional regulation

**DOI:** 10.1038/s41380-019-0529-7

**Published:** 2019-10-21

**Authors:** Joshua C. Bis, Xueqiu Jian, Brian W. Kunkle, Yuning Chen, Kara L. Hamilton-Nelson, William S. Bush, William J. Salerno, Daniel Lancour, Yiyi Ma, Alan E. Renton, Edoardo Marcora, John J. Farrell, Yi Zhao, Liming Qu, Shahzad Ahmad, Najaf Amin, Philippe Amouyel, Gary W. Beecham, Jennifer E. Below, Dominique Campion, Laura Cantwell, Camille Charbonnier, Jaeyoon Chung, Paul K. Crane, Carlos Cruchaga, L. Adrienne Cupples, Jean-François Dartigues, Stéphanie Debette, Jean-François Deleuze, Lucinda Fulton, Stacey B. Gabriel, Emmanuelle Genin, Richard A. Gibbs, Alison Goate, Benjamin Grenier-Boley, Namrata Gupta, Jonathan L. Haines, Aki S. Havulinna, Seppo Helisalmi, Mikko Hiltunen, Daniel P. Howrigan, M. Arfan Ikram, Jaakko Kaprio, Jan Konrad, Amanda Kuzma, Eric S. Lander, Mark Lathrop, Terho Lehtimäki, Honghuang Lin, Kari Mattila, Richard Mayeux, Donna M. Muzny, Waleed Nasser, Benjamin Neale, Kwangsik Nho, Gaël Nicolas, Devanshi Patel, Margaret A. Pericak-Vance, Markus Perola, Bruce M. Psaty, Olivier Quenez, Farid Rajabli, Richard Redon, Christiane Reitz, Anne M. Remes, Veikko Salomaa, Chloe Sarnowski, Helena Schmidt, Michael Schmidt, Reinhold Schmidt, Hilkka Soininen, Timothy A. Thornton, Giuseppe Tosto, Christophe Tzourio, Sven J. van der Lee, Cornelia M. van Duijn, Otto Valladares, Badri Vardarajan, Li-San Wang, Weixin Wang, Ellen Wijsman, Richard K. Wilson, Daniela Witten, Kim C. Worley, Xiaoling Zhang, Alzheimer’s Disease Sequencing Project, Celine Bellenguez, Jean-Charles Lambert, Mitja I. Kurki, Aarno Palotie, Mark Daly, Eric Boerwinkle, Kathryn L. Lunetta, Anita L. Destefano, Josée Dupuis, Eden R. Martin, Gerard D. Schellenberg, Sudha Seshadri, Adam C. Naj, Myriam Fornage, Lindsay A. Farrer

**Affiliations:** 1 Department of Medicine (General Internal Medicine), University of Washington, Seattle, WA USA; 2 Institute of Molecular Medicine, McGovern Medical School, University of Texas Health Science Center at Houston, Houston, TX USA; 3 John P. Hussman Institute for Human Genomics, Miller School of Medicine, University of Miami, Miami, FL USA; 4 Departments of Biostatistics, Boston University School of Public Health, Boston, MA USA; 5 Case Western Reserve University, Cleveland Heights, OH USA; 6 Human Genome Sequencing Center and Department of Molecular and Human Genetics, Baylor College of Medicine, Houston, TX USA; 7 Department of Medicine (Biomedical Genetics), Boston University School of Medicine, Boston, MA USA; 8 Department of Neuroscience and Ronald M Loeb Center for Alzheimer’s Disease, Icahn School of Medicine at Mount Sinai, New York, NY USA; 9 Department of Genetics and Genomics Sciences, Icahn School of Medicine at Mount Sinai, New York, NY USA; 10 University of Pennsylvania Perelman School of Medicine, Philadelphia, PA USA; 11 Erasmus University Medical Center, Rotterdam, Netherlands; 12 Inserm, U1167, RID-AGE-Risk Factors and Molecular Determinants of Aging-Related Diseases, Lille, France; 13 Institut Pasteur de Lille, Lille, France; 14 University Lille, U1167-Excellence Laboratory LabEx DISTALZ, Lille, France; 15 Department of Medical Genetics, Vanderbilt University Medical Center, Nashville, TN USA; 16 Department of Genetics and CNR-MAJ, Normandie Université, UNIROUEN, Inserm U1245 and Rouen University Hospital, F 76000, Normandy Centre for Genomic and Personalized Medicine, Rouen, France; 17 Department of Research, Centre Hospitalier du Rouvray, Sotteville-lès-, Rouen, France; 18 Department of Psychiatry, Washington University, St. Louis, MO USA; 19 National Heart, Lung, and Blood Institute’s Framingham Heart Study, Framingham, MA USA; 20 University of Bordeaux, Inserm, Bordeaux Population Health Research Center, team VINTAGE, UMR 1219, F-33000 Bordeaux, France; 21 Department of Neurology and Institute for Neurodegenerative Diseases, Bordeaux University Hospital, Memory Clinic, F-33000 Bordeaux, France; 22 Centre National de Recherche en Génomique Humaine, Institut François Jacob, Direction de le Recherche Fondamentale, CEA, Evry, France; 23 McDonnell Genome Institute, Washington University, St. Louis, MO USA; 24 Broad Institute of MIT and Harvard, Cambridge, MA USA; 25 Inserm UMR-1078, CHRU Brest, Université Brest, Brest, France; 26 Institute for Molecular Medicine Finland (FIMM), University of Helsinki, Helsinki, Finland; 27 National Institute for Health and Welfare, Helsinki, Finland; 28 Institute of Clinical Medicine - Neurology and Department of Neurology, University of Eastern Finland, Kuopio, Finland; 29 Institute of Biomedicine, University of Eastern Finland, Kuopio, Finland; 30 Program in Medical and Population Genetics and Genetic Analysis Platform, Stanley Center for Psychiatric Research, Broad Institute of MIT and Harvard, Cambridge, MA USA; 31 Psychiatric & Neurodevelopmental Genetics Unit, Massachusetts General Hospital, Boston, MA USA; 32 McGill University and Génome Québec Innovation Centre, Montréal, Canada; 33 Department of Clinical Chemistry, Fimlab Laboratories and Finnish Cardiovascular Research Center-Tampere, Faculty of Medicine and Life Sciences, University of Tampere, Tampere, Finland; 34 Department of Medicine (Computational Biomedicine), Boston University School of Medicine, Boston, MA USA; 35 Columbia University, New York, NY USA; 36 Indiana University School of Medicine, Indianapolis, IN USA; 37 University of Tartu, Estonian Genome Center, Tartu, Estonia; 38 Department of Epidemiology, University of Washington, Seattle, WA USA; 39 Department of Health Services, University of Washington, Seattle, WA USA; 40 Kaiser Permanente Washington Health Research Institute, Seattle, WA USA; 41 Inserm, CNRS, Univ. Nantes, CHU Nantes, l’institut du thorax, Nantes, France; 42 Unit of Clinical Neuroscience, Neurology, University of Oulu and Medical Research Center, Oulu University Hospital, Oulu, Finland; 43 Department of Neurology, Clinical Division of Neurogeriatrics, Medical University of Graz, Graz, Austria; 44 Department of Neurology, Kuopio University Hospital, Kuopio, Finland; 45 Department of Statistics, University of Washington, Seattle, WA USA; 46 Department of Medicine (Medical Genetics), University of Washington, Seattle, WA USA; 47 Department of Biostatistics, University of Washington, Seattle, WA USA; 48 School of Public Health, University of Texas Health Science Center at Houston, Houston, TX USA; 49 Departments of Neurology, Boston University School of Medicine, Boston, MA USA; 50 Glenn Biggs Institute for Alzheimer’s and Neurodegenerative Diseases, University of Texas Health Sciences Center, San Antonio, TX USA; 51 Department of Epidemiology, Boston University School of Public Health, Boston, MA USA; 52 Department of Ophthalmology, Boston University School of Medicine, Boston, MA USA

**Keywords:** Genetics, Diseases

**Correction to: Molecular Psychiatry**

10.1038/s41380-018-0112-7 published online 14 August 2018

Following publication, the authors noticed that ‘Laura Cantwell’, ‘Otto Valladares’, and ‘Li-San Wang’ were inadvertently omitted from the author list. These authors have now been added to the author list in 21st, 77th, and 79th position, respectively. This has been corrected in both the PDF and HTML versions of the article.

