# Supplementary material for: Whole exome sequencing study identifies novel rare and common Alzheimer’s-Associated variants involved in immune response and transcriptional regulation
Source: Mol Psychiatry. 2018 Aug 14;25(8):1859–75. doi: 10.1038/s41380-018-0112-7 (PMC6375806; doi:10.1038/s41380-018-0112-7)
Supplement: Supplementary file 1 — Supplementary tables and figures [file 41380_2018_112_MOESM1_ESM.docx]

**APPENDIX CONTENTS**

**Supplementary Methods**

**Table S1.** Characteristic of the Cohorts for Heart and Aging Research in Genomic Epidemiology (CHARGE) Datasets.

**Table S2.** Characteristic of Alzheimer’s Disease Genetics Consortium (ADGC) Datasets.

**Figure S1.** Plot of first and second principal components (PCs) from EIGENSTRAT.

**Figure S2.** Correlation of association test results among logistic regression models.

**Figure S3.** Variant allele frequency by predicted impact

**Figure S4**. QQ Plots for Individual Variants Meta Analyses

**Figure S5.** QQ Plots for SKAT-O (moderate or high impact variants)

**Figure S6.** QQ Plots for SKAT-O (high impact variants)

**Figure S7.** QQ Plots for SKAT-O (loss-of-function variants)

**Figure S8.** Correlation of VEP annotation pipeline and CADD score results.

**SUPPLEMENTARY TABLES (in excel spreadsheet)**

**Table S3.** Association results for single nucleotide variants and short indels.

**Table S4.** Gene-based association results

**Table S5.** Gene-based association results using CADD annotation.

**Table S6.** Replication analysis results

**Table S7.** Association results for *STAG3* and *ZNF655* conditioned on *PILRA*

**Table S8.** Segregating top-ranked variants from WES discovery sample in ADSP whole genome sequence family-based dataset.

**Table S9.** Gene-based results for top-ranked genes from WES discovery sample in ADSP whole genome sequence family-based dataset.

**Table S10.** High and moderate impact variants in previously established AD genes

**SUPPLEMENTARY METHODS**

**Processing Whole Exome Sequence Data**

The ADSP Quality Control (QC) Working Group performed quality control using a protocol developed for WGS data with additional and modified filtering steps, including (1) filtering to include only variants overlapping the target capture regions of the two capture kits used by the three sequencing centers (Illumina and Nimblegen), and (2) filtering on extreme departure from Hardy-Weinberg equilibrium (HWE, *P*<10^-6^) for unrelated controls.^1^ This QC protocol also excluded monomorphic variants, variants with call rates <80%, and variants with low average read depth (<10 reads). Post-hoc QC filtering implemented by the Case-Control Working Group included removing samples with a high degree of cryptic relatedness with other samples, population substructure outlier samples, and samples with sex mismatches.

**Cryptic Relatedness**

Although participants were intended to be unrelated across multiple cohorts, we investigated cryptic relatedness using pairwise identity-by-descent (IBD) estimation as implemented in PLINK.[^2^](#_ENREF_28)  A subset of 55,937 SNVs with a minor allele frequency (MAF)>0.01 and call rate>95% underwent ‘LD pruning’ (filtering on the basis of linkage disequilibrium (LD) to remove highly correlated SNVs), resulting in a set of 37,793 SNVs used in IBD estimation. Pairs of individuals with $\hat{\pi}$ (proportion of alleles shared IBD)>0.2 were considered related for the purposes of characterizing population substructure as described below. For association analyses, pairs of individuals with $\hat{\pi}$>0.4 were identified and one sample per pair was excluded, with inclusion prioritizing ‘enriched’ over other AD cases, as well as AD cases with an autopsy-based diagnosis. We identified 69 participants as related to other participants due to high IBD and excluded them from subsequent analyses.

**Population Substructure**

Prior to association analyses, we performed principal components analyses (PCA) in EIGENSTRAT[^3,4^](#_ENREF_29) using the *smartpca* program to (1) determine ethnicity/race for samples with missing ancestry information; (2) identify outliers among participants with self-reported EA ancestry; and (3) compute principal components (PCs) to adjust for population structure in association analyses. We selected SNVs based on these filtering criteria: MAF≥5%, call rate≥99%, and only one SNV for each pair of SNVs with linkage disequilibrium (LD) of *r^2^*>0.5 in a 50-SNV window. ADSP participants with individual call rate<90% were excluded, and only one participant from every pair with estimated $\hat{\pi}$>0.2 was selected.

We computed PCs for self-reported EA individuals and individuals with missing ancestry information using the 1000 Genomes (1000G) Phase 3 reference panel[^5^](#_ENREF_31) and a subset of 12,351 SNVs that met the above criteria and were found in both ADSP and 1000G subjects. PCs were computed using all unrelated ADSP and 1000G subjects and projected on individuals that were omitted from the PC computation due to high IBD or low call rate. ADSP participants falling within six standard deviations of the mean PC value for CEU/TSI 1000G subjects for the first four PCs were confirmed or identified as EA. Among 10,444 self-reported EA participants and 69 participants with missing self-reported ethnicity information, 10,480 were identified as EA and 33 were excluded as outliers **(Supplementary Figure S1**). Those who self-reported as Hispanic (N=395) were considered CH for these analyses. To compute PCs for covariate adjustment, we used 12,351 SNVs satisfying the above criteria in all ADSP participants, computed separately for EA and CH participants, and assigned derived values to any related participants ($\hat{\pi}$>0.2) or samples with low call rate.

### **Differential Missingness**

### Differential missingness between cases and controls was tested separately by ancestry using PLINK v1.9 using the “midp” correction (‘--test-missing midp’). We excluded variants with differential missingness of *P*<10^-6^ in stratum-specific analyses.

**Variant annotation**

Variants were assigned to one or more genes using the VEP annotation pipeline[^6^](#_ENREF_32) and SnpEff.^7^ Variants with multiple functional annotations were classified according to the annotation predicted to be the most damaging within each assigned gene using the following categories:

1. HIGH IMPACT: variants classified by VEP or SnpEff as splice acceptor, splice donor, stop gained, frameshift, stop lost, start lost, or transcript amplification;
2. HIGH or MODERATE IMPACT: included the categories above plus variants annotated by VEP or SnpEff as inframe insertion, inframe deletion, missense variant, or protein altering.
3. LOSS-OF-FUNCTION classifications made use of LOFTEE (<https://github.com/konradjk/loftee>), a VEP plugin implemented in the WGSA pipeline.[^8^](#_ENREF_35) Further, the following criteria were applied to filter out low-confidence LoF variants: (1) splice site variants in small introns or in an intron with non-canonical splice site; (2) stop-gained variants in the last 5% of the transcript or in an exon with non-canonical splice sites around it; and (3) all variants of which the LoF allele was the ancestral state.
4. CADD scores > 15 which corresponds to the median value for all possible canonical splice site changes and non-synonymous variants and CADD scores > 20 which are equivalent to the 1% most deleterious variants.[^9^](#_ENREF_36)

**Descriptions of Replication Datasets**

**FinnAD WES Dataset**

The FinnAD exome sequencing study dataset included 1,024 Finnish cases between the ages of 60 and 90 years and 4,322 Finnish population controls who were non-demented and older than 60 years and did not show signs of cognitive impairment when last assessed or at death. All cases and controls were unrelated European ancestry individuals. Cases were recruited from four different studies: Kuopio AD, Tampere AD, Finnish Twin Cohort study and FINRISK. All controls were from the FINRISK study. Kuopio AD is a clinic based collection of AD patients examined in the Department of Neurology in Kuopio University Hospital. All patients were diagnosed with probable AD according to the National Institute of Neurological and Communicative Disorders and Stroke and the Alzheimer’s Disease and Related Disorders Association (NINCDS-ADRDA) criteria.[^10^](#_ENREF_1) Tampere AD cases were examined in the Department of Neurology, Tampere University Hospital Finland and diagnosed using NINCDS-ARDARA criteria as previously described.[^11^](#_ENREF_2) AD cases were also identified from the Finnish Twin Cohort study[^12^](#_ENREF_3) by using combination of Finnish cause of death registries and TELE/TICS interviews.[^13^](#_ENREF_4) FINRISK is a series of population-based health examination surveys carried out every five years since 1972 to monitor the risk of chronic diseases, as detailed elsewhere.[^14^](#_ENREF_5) The cohorts have been followed up for Alzheimer and other disease end-points using annual record linkage with the Finnish National Hospital Discharge Register, the National Causes-of-Death Register and the National Drug Reimbursement Register as described in Tynkkynen *et al.* 2017.[^15^](#_ENREF_6)

FINRISK exomes were sequenced at McDonnell Genome Institute at Washington University using Roche SeqCap EZ HGSC VCRome and sequenced using Illumina HiSeq2000 or 2500 and the other AD samples were exomes were sequenced at the Broad Institute using Illumina nextera Rapid Capture Exome-capture kit and sequenced with Illumina HiSeq2000 or 2500. All samples were called jointly with other Finnish samples from Sequencing Initiative Suomi (sisuproject.fi) using Broad Institute’s standard GATK HaplotypeCaller variant calling pipeline as described elsewhere.[^16^](#_ENREF_7) We removed samples that were: population outliers, outliers in terms of percent chimeras, insertion/deletion-ratio, het/hom var-ratio and number of singletons. We set all genotypes to missing if depth<10, GQ<20 or heterozygote allelic balance out of 20%/80%-ratio of referent and alternate allele reads respectively. We removed variants which met one or more of the following criteria: VQSR non-pass, genotype missingess ≤0.9 in cases or controls, not in Hardy-Weinberg equilibrium (*P≤*10^-6^) among controls, SNP qual-by-depth <2, INDEL qual-by-depth <3, or more than 20% of heterozygote genotypes deviating from 80/20 allelic balance.

**ADES-FR WES dataset**

The ADES-FR project combines WES and WGS data from 1,779 AD cases and 1,273 controls, all originating from France. Details on the sequencing and quality control of the data are described elsewhere.[^17^](#_ENREF_8) Briefly, exomes of 2,103 individuals were captured using the Agilent SureSelect All Exons Human V1, V2, V3, V4, V4+UTR, V5 or V5+UTR kits, while WGS sequencing was performed on 955 individuals. Read alignment and variant calling were performed in 4 different centers using similar bioinformatics pipelines. In particular, variants were called with GATK. Genotypes with genotype quality below 20 were set as missing. Sample quality control was performed on the following criteria: missingness, heterozygosity/homozygosity ratio (HetHom ratio), transition-transversion ratio (Ti/Tv), sex mismatches, outlying ancestry, relatedness and number of private mutations. For this specific analysis, we further excluded cases with age at onset below 60 or above 90 and controls with age below 60, leading to a final sample size of 1,142 AD cases and 1,104 controls. Multi-allelic sites were decomposed into bi-allelic variants and normalized. Variants were filtered according to their VQSLOD value, computed by the Variant Quality Score Recalibration (VQSR) step of GATK, using a sensitivity threshold of 99.5% for SNVs and 99% for indels. Variants with missingness >5%, Hardy-Weinberg Equilibrium *P*<10^-7^ in controls and outlying allele balance values (ABHet >0.75, ABHet <0.25 or ABHom <0.9) were excluded from the analyses. We also excluded variants in low-complexity regions and variants in short tandem repeat regions. Besides, multi-allelic sites where more than one alternate allele had a frequency below the frequency of the reference allele were excluded. Finally, variants with differential missingness between cases and controls of *P*<10^-7^ were excluded. After QC, a total of 1,142 cases and 1,104 controls remained for analysis.

Association tests were performed with the R package seqMeta, with an adjustment for the first three principal components of ancestry, age, and sex in Model 1, and additionally for *APOE* ε4 and ε2 dosages in Model 2.

**CHARGE WES dataset**

We undertook replication in a sample of Alzheimer disease (AD) cases compared to a sample of cognitively normal controls drawn from persons enrolled in the same CHARGE cohorts that are included within the ADSP Discovery sample. Thus, cases and controls were both drawn from the Atherosclerosis Risk in Communities study (ARIC), the Cardiovascular Health Study (CHS), the Framingham Heart Study (FHS) and the Rotterdam study (RS). These participants had whole exome sequencing done through alternative (non-ADSP) funding sources, and were not selected to be included as either a case or a control through the ADSP sampling frame.

All cases met the same diagnostic criteria as the cases included in the ADSP discovery samples. They were all older than age 60 years and met NINCDS-ADRDA criteria for probable, possible or definite (autopsy-confirmed) AD. We excluded persons with dementia that was not primarily attributed to AD. In addition, we excluded persons in whom the onset of clinical dementia occurred after age 90 years as the etiology of dementia was more likely to be multi-factorial in these oldest-old individuals.

Controls were also required to be over age 60 years and to have some cognitive assessment that documented absence of clinical dementia at the time of their selection. We decided to include 3 times as many controls as cases as a ratio of 1:3 cases to controls optimizes power for association analyses. These controls were selected by random sampling of all eligible controls. Age was defined for cases as age at initial diagnosis of dementia due to AD and for controls as age at which they were last known to be alive and free of dementia or to have died free of clinical dementia.

The distribution of cases and controls across the 4 cohorts is shown in **Supplementary Table S1**. We had 612 late-onset AD cases (mean age = 81.5) and 1,836 dementia-free controls (mean age = 80.3). Only persons of European ancestry were included since the minority profile varied between the ADSP and CHARGE samples. The ADSP Discovery samples had no individuals of African ancestry whereas the CHARGE samples have no participants of Caribbean-Hispanic ancestry. Whole exome sequencing was undertaken in these persons through various projects, including the CHARGE-S grant (PI: Boerwinkle), the Center Initiated Project (CIP; PI: Boerwinkle), and various WES projects at the Erasmus University, Rotterdam (PI: van Duijn). The CHARGE cohorts' study participants included here were selected as part of a large random cohort sample or for extreme values for at least one of the following phenotypes: age at menopause, electrocardiogram QT interval, fasting blood glucose, fibrinogen level, renal function, extremes of cardiovascular disease risk factors selected by principle components (PCs), and waist-to-hip ratio. Participants were selected for sequencing through the CIP based on the availability of at least two brain MRI measures or two measures of cognitive change for each person.

Details of sequencing of these samples and variant calling have been previously published.[^18^](#_ENREF_9) All sequences were called together at the Baylor College of Medicine using the ATLAS2 pipeline and a common SNP information file including all genetic variation observed across all 4 cohorts was created. This annotation file included quality-controlled variants observed in at least one cohort. All novel variants identified in the CHARGE replication dataset were annotated by the ADSP annotation working group using identical methods to identify putative functional as those used for the ADSP Discovery analyses. In summary, moderate to high predicted effect variants were identified based on Variant Effect Predictor and loss of function variants as predicted by dbNSFP v2.0 according to GRCh37 and RefSeq. This multiple study SNPinfo file was used as a component of the R package seqMeta.^19^

All statistical analyses were implemented using the seqMeta R package and Bonferroni corrections were applied. Each cohort ran its own cohort-specific analysis. Two logistic regression models were run using one model adjusted only for age-, sex- and principal components (CHARGE replication model 1) and a second model additionally adjusted for absence or presence of an *APOE* ε4 allele (CHARGE replication model 2). In the CHARGE replication sample the ages of cases and controls were more closely matched than in the ADSP discovery sample; hence an analogous model to the ADSP Model 0 analysis (with adjustment only for principal components) was not run. We examined associations with case or control status for single nucleotide variants (SNV) and indels individually, and using sets of rare variants grouped by gene locations and variant properties using the same variant selection criteria as for the ADSP Discovery analyses; we used SKAT-O for gene based analyses.

Model specific results were pooled across the 4 cohorts using inverse variance meta-analyses. Results for the genes of interest were extracted and results for the CHARGE replication Model 1 were pooled with results for ADSP Discovery Models 0 and 1. Results for CHARGE replication Model 2 were pooled with results for ADSP discovery Model 2.

**ADGC GWAS dataset (Imputed to HRC r1.1)**

Replication was performed using genotypes from 33 ADGC datasets. Genotyping arrays that were used for most datasets were described in detail elsewhere,[^20,^](#_ENREF_10)^21^ except for the Chicago Health and Aging Project (CHAP), Netherlands Brain Bank (NBB), Texas Alzheimer’s Research and Care Consortium (TARCC), and Washington Heights/Inwood Columbia Aging Project (WHICAP) datasets. CHAP and WHICAP datasets were genotyped on the Illumina OmniExpress-24 array, and NBB was genotyped on the Illumina 1M platform. TARCC first wave subjects were genotyped using the Affymetrix 6.0 microarray chip, while subjects in the second wave (172 cases and 74 controls) were genotyped using the Illumina HumanOmniExpress-24 beadchip. Second wave TARCC subjects (TARCC2) were genotyped together with 84 cases and 115 controls from second wave samples ascertained at the University of Miami and Vanderbilt University. All samples used in stage 3 were imputed to the HRC haplotype reference panel,^22,23^ which includes 64,976 haplotypes with 39,235,157 SNPs that allows imputation down to an unprecedented MAF=0.00008.

Prior to imputation, all genotype data underwent QC procedures that have been described extensively elsewhere.[^20,21^](#_ENREF_10) Imputation was performed on the Michigan Imputation Server (<https://imputationserver.sph.umich.edu/>) running MiniMac3.[^24^](#_ENREF_14) Genotypes from genome-wide, high-density SNP genotyping arrays for 16,175 AD cases and 17,176 cognitive-normal individuals were imputed. Across all samples 39,235,157 SNPs were imputed, with the actual number of SNPs imputed for each individual varying based on the regional density of array genotypes available. Because a subset of these samples had also been genotyped as part of stage 1, we examined the imputation quality for critical variants by comparing imputed genotypes to those directly genotyped by the exome array; overall concordance was >99%, while concordance among alternate allele genotypes (heterozygotes and alternate allele homozygotes) was >88.5% on average (N~13,000 samples).

All datasets where filtered to exclude ADGC samples already incorporated into ADSP, and included a total of 9,841 cases and 11,697 cognitively normal elderly individuals for inclusion in replication analyses. The characteristics of these samples are provided in **Supplementary Table S2**.

We performed single-variant association analyses using logistic regression on imputed genotype dosages assuming an additive Mendelian model of inheritance, with covariate adjustment for the first three principal components adjusting for population substructure (derived using EIGENSTRAT [^3,4^](#_ENREF_16)). Principal components were re-estimated using only the samples which did not overlap with the ADSP sample. Association on each dataset was performed using PLINK[^2^](#_ENREF_17) for each case-control dataset, and the “glmm” package in R for the LOAD and MIRAGE family-based datasets. Fixed-effects meta-analysis across all datasets was done using the program METAL. Association for low-frequency variants (MAF<0.02) was also performed using the SeqMeta/R package.^19,25^

Gene-based association tests estimated the aggregate effect of risk and protective variants within a “gene” region as defined by gene annotations. We performed gene-based tests using SKAT-O, which optimally combines SKAT and burden tests, as implemented in the SeqMeta/R. For these analyses, the SKAT portion of the test included variants with a minor allele frequency (MAF) ≤0.05; the burden component aggregated variants with MAF ≤0.01. The SKAT test used ‘Wu weights’, defined by a beta density function with pre-specified parameters a1=1 and a2=25, evaluated at the sample minor allele frequency. The SKAT-O statistic, a linear combination between a SKAT statistic (Q_skat_) and a burden statistic (Q_burden_) equal to (1-ρ) Q_skat_ + ρQ_burden_, was optimized across 11 values of ρ (0.1 increments), and calculation of the significance took into consideration the multiple values of ρ evaluated.

**References**

1. Naj AC, Lin H, Vardarajan BN, et al. Quality control and integration of genotypes from two calling pipelines for whole genome sequence data in the Alzheimer’s Disease Sequencing Project. *Genomics* 2018. In press.

2. Purcell S, Neale B, Todd-Brown K, et al. PLINK: a tool set for whole-genome association and population-based linkage analyses. *Am J Hum Genet* 2007; 81:559-575.

3. Price AL, Patterson NJ, Plenge RM, Weinblatt ME, Shadick NA, Reich D. Principal components analysis corrects for stratification in genome-wide association studies. *Nat Genet* 2006; 38:904-909.

4. Patterson N, Price AL, Reich D. Population structure and eigenanalysis. *PLoS Genet* 2006; 2:e190.

5. Genomes Project Consortium, Auton A, Brooks LD, Durbin RM, et al. A global reference for human genetic variation. *Nature* 2015; 526:68-74.

6. McLaren W, Gil L, Hunt SE, et al. The Ensembl Variant Effect Predictor. *Genome Biol* 2016; 17:122.

7. Cingolani P, Platts A, Wang le L, et al. A program for annotating and predicting the effects of single nucleotide polymorphisms, SnpEff: SNPs in the genome of Drosophila melanogaster strain w1118; iso-2; iso-3. *Fly (Austin)* 2012; 6:80-92

8. Liu X, White S, Peng B, et al. WGSA: an annotation pipeline for human genome sequencing studies. *J Med Genet* 2016; 53:111-112.

9. Kircher M, Witten DM, Jain P, O'Roak BJ, Cooper GM, Shendure J. A general framework for estimating the relative pathogenicity of human genetic variants. *Nat Genet* 2014; 46:310-315.

10. McKhann G, Drachman D, Folstein M, Katzman R, Price D, Stadlan EM. Clinical diagnosis of Alzheimer's disease: report of the NINCDS-ADRDA Work Group under the auspices of Department of Health and Human Services Task Force on Alzheimer's Disease. *Neurology* 1984:34:939-944.

11. Lehtimaki T, Pirttila T, Mehta PD, Wisniewski HM, Frey H, Nikkari T. Apolipoprotein E (apoE) polymorphism and its influence on ApoE concentrations in the cerebrospinal fluid in Finnish patients with Alzheimer's disease. *Hum Genet* 1995; 95:39-42.

12. Kaprio J. The Finnish Twin Cohort Study: an update. *Twin Res Hum Genet* 2013; 16:157-162.

13. Vuoksimaa E, Rinne JO, Lindgren N, Heikkila K, Koskenvuo M, Kaprio J. Middle age self-report risk score predicts cognitive functioning and dementia in 20-40 years. *Alzheimer Dement* 2016; 4:118-125.

14. Borodulin K, Vartiainen E, Peltonen M, et al. Forty-year trends in cardiovascular risk factors in Finland. *Eur J Pub Health* 2015; *25*:539-546.

15.Tynkkynen J, Hernesniemi JA, Laatikainen T, et al. High-sensitivity cardiac troponin I and NT-proBNP as predictors of incident dementia and Alzheimer's disease: the FINRISK Study. *J Neurol* 2017; 264:503-511.

16. Rivas MA, Graham D, Sulem P, et al. A protein-truncating R179X variant in RNF186 confers protection against ulcerative colitis. *Nat Comm* 2016: 7:12342.

17. Bellenguez C, Charbonnier C, Grenier-Boley B, et al. (Contribution to Alzheimer's disease risk of rare variants in TREM2, SORL1, and ABCA7 in 1779 cases and 1273 controls. *Neurobiol Aging* 2017; 59:220 e221-220 e229.

18. Yu B, Pulit SL, Hwang SJ, et al. Rare exome sequence variants in CLCN6 reduce blood pressure levels and hypertension risk. *Circul Cardiovasc Genet* 2016; 9:64-70.

19. Lumley T, Brody J, Dupuis J, Cupples A. Meta-analysis of a rare-variant association test: University of Auckland; 2012. http://stattech.wordpress.fos.auckland.ac.nz/files/2012/11/skat-meta-paper.pdf. Technical report.

20. Naj AC, Jun G, Beecham GW, et al. Common variants at MS4A4/MS4A6E, CD2AP, CD33 and EPHA1 are associated with late-onset Alzheimer's disease. *Nat Genet* 2011; 43:436-441.

21. Lambert JC, Ibrahim-Verbaas CA, Harold D, et al. Meta-analysis of 74,046 individuals identifies 11 new susceptibility loci for Alzheimer's disease. *Nat Genet* 2013; 45:1452-1458.

22. Das S, Forer L, Schonherr S, et al. Next-generation genotype imputation service and methods. *Nat Genet* 2016; 48:1284-1287.

23. McCarthy S, Das S, Kretzschmar W, et al. A reference panel of 64,976 haplotypes for genotype imputation. *Nat Genet* 2016; 48:1279-1283.

24. Howie B, Fuchsberger C, Stephens M, Marchini J, Abecasis GR. Fast and accurate genotype imputation in genome-wide association studies through pre-phasing. *Nat Genet* 2012; 44:955-959.

25. Lee S, Emond MJ, Bamshad MJ, et al. Optimal unified approach for rare-variant association testing with application to small-sample case-control whole-exome sequencing studies. *Am J Hum Genet* 2012; 91:224-237.

**Supplementary Table S1.** Characteristics of the Cohorts for Heart and Aging Research in Genomic Epidemiology (CHARGE) datasets

| Dataset | AD Cases | | |  | Cognitively Normal Controls | | |
| --- | --- | --- | --- | --- | --- | --- | --- |
|  | N | Age  (mean) | Sex  (%F) |  | N | Age  (mean) | Sex  (%F) |
| ARIC | 68 | 80 | 63% |  | 204 | 76 | 64% |
| CHS | 190 | 81 | 66% |  | 570 | 79 | 62% |
| FHS | 39 | 80 | 64% |  | 117 | 73 | 50% |
| RS | 315 | 82 | 68% |  | 945 | 83 | 55% |
| TOTAL | 612 | -- | -- |  | 1,836 | -- | -- |

**Supplementary Table S2.** Characteristic of Alzheimer’s Disease Genetics Consortium (ADGC) datasets.

| Dataset | AD Cases (N=9,841) | | | |  | Cognitively Normal Controls (N=11,697) | | | |
| --- | --- | --- | --- | --- | --- | --- | --- | --- | --- |
|  | N | Age  (mean) | Sex (%F) | APOE E4  (% carrier) |  | N | Age  (mean) | Sex (%F) | APOE E4  (% carrier) |
| ACT1 | 258 | 80.1 | 64.3 | 77.9 |  | 709 | 77.2 | 56.8 | 29.2 |
| ACT2 | 13 | 82.5 | 69.2 | 76.9 |  | 3 | 81.3 | 66.7 | 0 |
| ADC1 | 631 | 73.8 | 59.1 | 85.9 |  | 306 | 71.9 | 63.7 | 39.2 |
| ADC2 | 326 | 74.3 | 55.2 | 75.2 |  | 132 | 74.4 | 67.4 | 31.9 |
| ADC3 | 529 | 73.7 | 56.7 | 74 |  | 480 | 72.8 | 63.8 | 27.5 |
| ADC4 | 106 | 74.0 | 58.5 | 75.8 |  | 312 | 73.8 | 64.7 | 31.3 |
| ADC5 | 124 | 75.4 | 63.7 | 83.8 |  | 344 | 73.1 | 65.1 | 28.5 |
| ADC6 | 74 | 75.0 | 63.5 | 78.6 |  | 262 | 71.2 | 65.7 | 31.9 |
| ADC7 | 514 | 72.5 | 51.4 | 62.9 |  | 788 | 73.9 | 63.7 | 32.2 |
| ADNI | 268 | 75.3 | 42.2 | 67.9 |  | 173 | 78.6 | 40.5 | 25.4 |
| BIOCARD | 6 | 73.8 | 33.3 | 50.0 |  | 111 | 67.9 | 62.2 | 32.4 |
| CHAP | 7 | 80.4 | 85.7 | 100 |  | 62 | 76.1 | 53.2 | 41 |
| EAS | 9 | 85.2 | 44.4 | 22.2 |  | 141 | 84.4 | 41.1 | 24.1 |
| GSK | 666 | 74.6 | 56.9 | 63.8 |  | 712 | 74.2 | 63.9 | 23.9 |
| LOAD | 433 | 73.3 | 66.5 | 79.7 |  | 645 | 74.3 | 62.3 | 33 |
| MAYO | 438 | 73.6 | 61.6 | 72.8 |  | 951 | 72.7 | 50.5 | 30.2 |
| MIRAGE | 150 | 72.2 | 65.3 | 63.3 |  | 249 | 72.3 | 59.8 | 37.6 |
| NBB | 80 | 74.5 | 71.3 | 58.8 |  | 48 | 81.5 | 56.3 | 16.7 |
| OHSU | 132 | 85.9 | 62.1 | 43.5 |  | 153 | 83.9 | 54.9 | 15.9 |
| PFIZER | 696 | 73.7 | 53.7 | 68.3 |  | 762 | 77.2 | 54.1 | 21 |
| RMAYO | 13 | 78.5 | 23.1 | 50.0 |  | 233 | 79.2 | 41.6 | 22 |
| ROSMAP1 | 123 | 85.5 | 74.0 | 80.9 |  | 380 | 78.3 | 73.2 | 22.3 |
| ROSMAP2 | 59 | 81.9 | 78.0 | 36.7 |  | 217 | 80.8 | 76.0 | 20.6 |
| TARC1 | 318 | 74.0 | 61.3 | 62.6 |  | 180 | 73.8 | 65.6 | 26.1 |
| TGEN2 | 665 | 73.5 | 64.8 | 65.3 |  | 362 | 80.0 | 48.3 | 21.4 |
| UKS | 594 | 72.2 | 57.6 | 55.7 |  | 170 | 64.1 | 51.2 | 14.3 |
| UMVUMSSM | 807 | 75.2 | 66.5 | 62.1 |  | 1,083 | 73.6 | 62.2 | 23.1 |
| UMVUTARC2 | 128 | 77.2 | 63.3 | 63.0 |  | 179 | 70.7 | 61.5 | 20 |
| UPITT | 1,254 | 72.9 | 62.9 | 58.0 |  | 826 | 75.5 | 63.3 | 19.8 |
| WASHU1 | 339 | 74.2 | 57.2 | 54.3 |  | 186 | 76.8 | 60.8 | 26.9 |
| WASHU2 | 38 | 73.4 | 57.9 | 56.8 |  | 94 | 71.4 | 46.8 | 26.6 |
| WHICAP | 43 | 84.2 | 74.4 | 34.9 |  | 444 | 80.3 | 61.7 | 23.3 |
| **TOTAL** | **9,841** | **74.4** | **60.1** | **66.3** |  | **11,697** | **75.1** | **59.8** | **26.7** |

**Supplementary Figure S1.** Plot of the first and second principal components (PCs) from EIGENSTRAT including all 10,909 ADSP WES case-control samples and 1000G Phase 3 multi-ethnic reference panel samples, included to confirm self-reported ethnicity and identify population outliers.


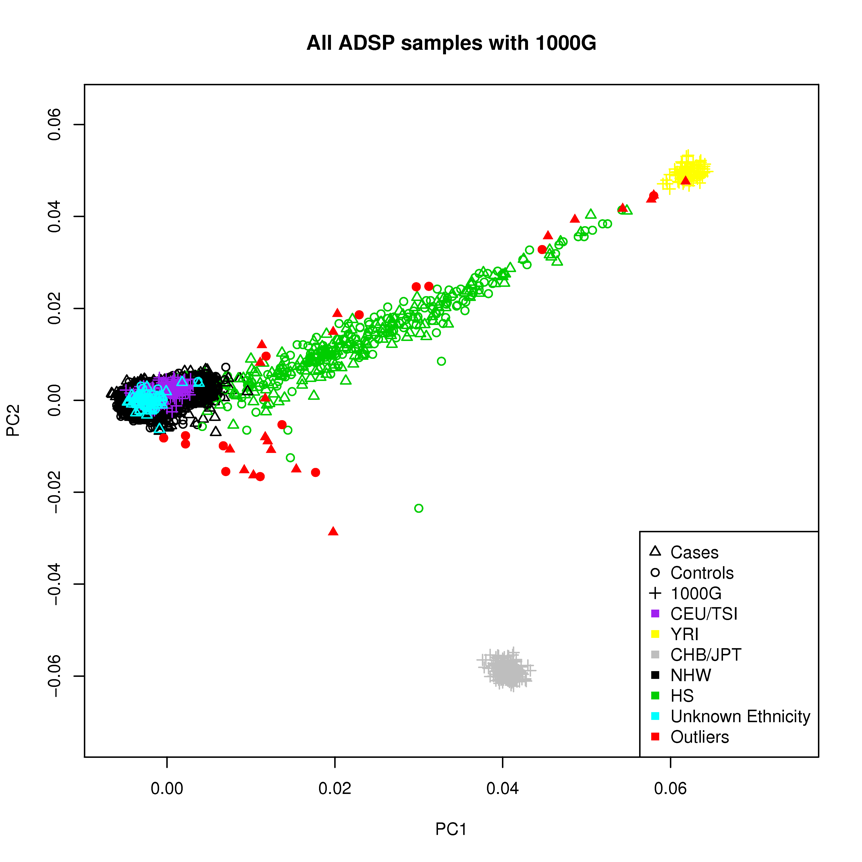


**Supplementary Figure S2.** Correlation of association test results among logistic regression models. Z-scores were calculated as B/SE for each of the main models and plotted against each of the other models. Numbers represent Pearson correlation coefficients. Green dots on scatterplots represent variants on within +/- 500kb of *APOE*.

| **Comparisons of Models 0, 1, 2** | **Comparisons of Models 0, 1, 2**  **(also includes the EA-only analyses)** |
| --- | --- |
| **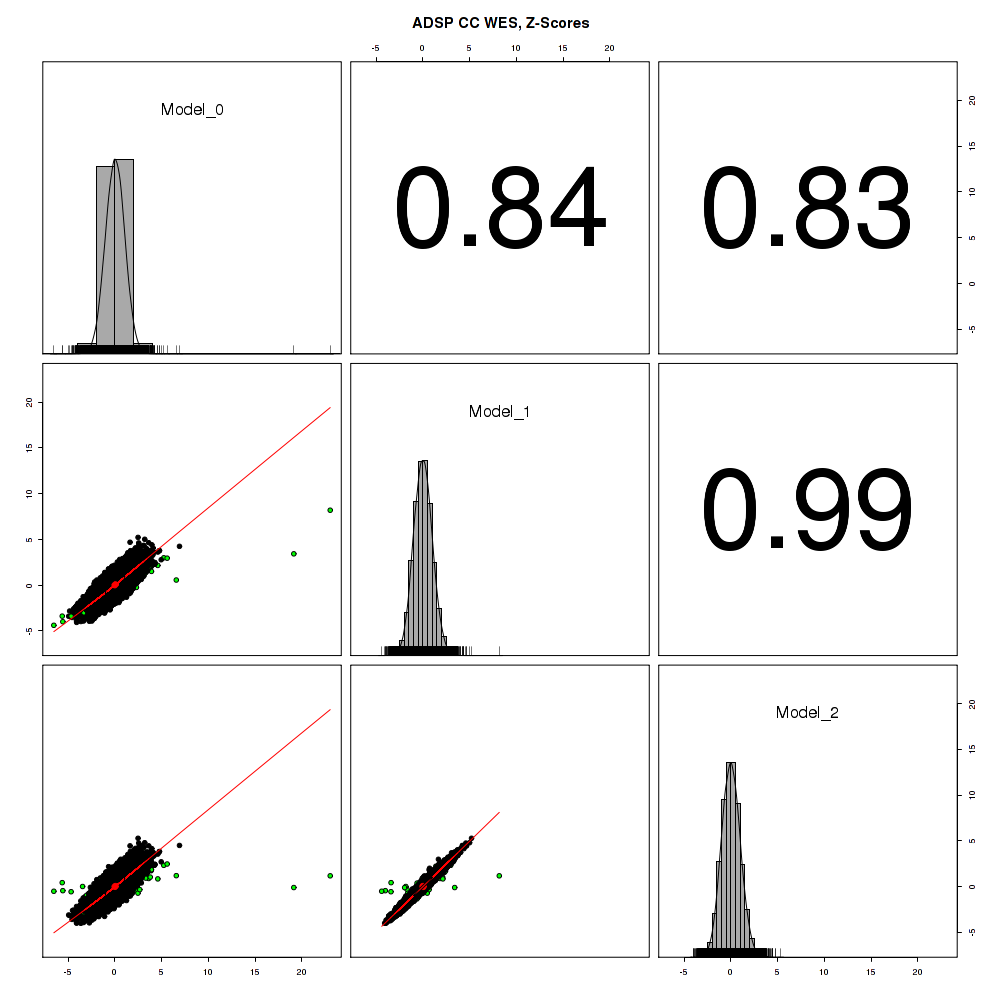** | **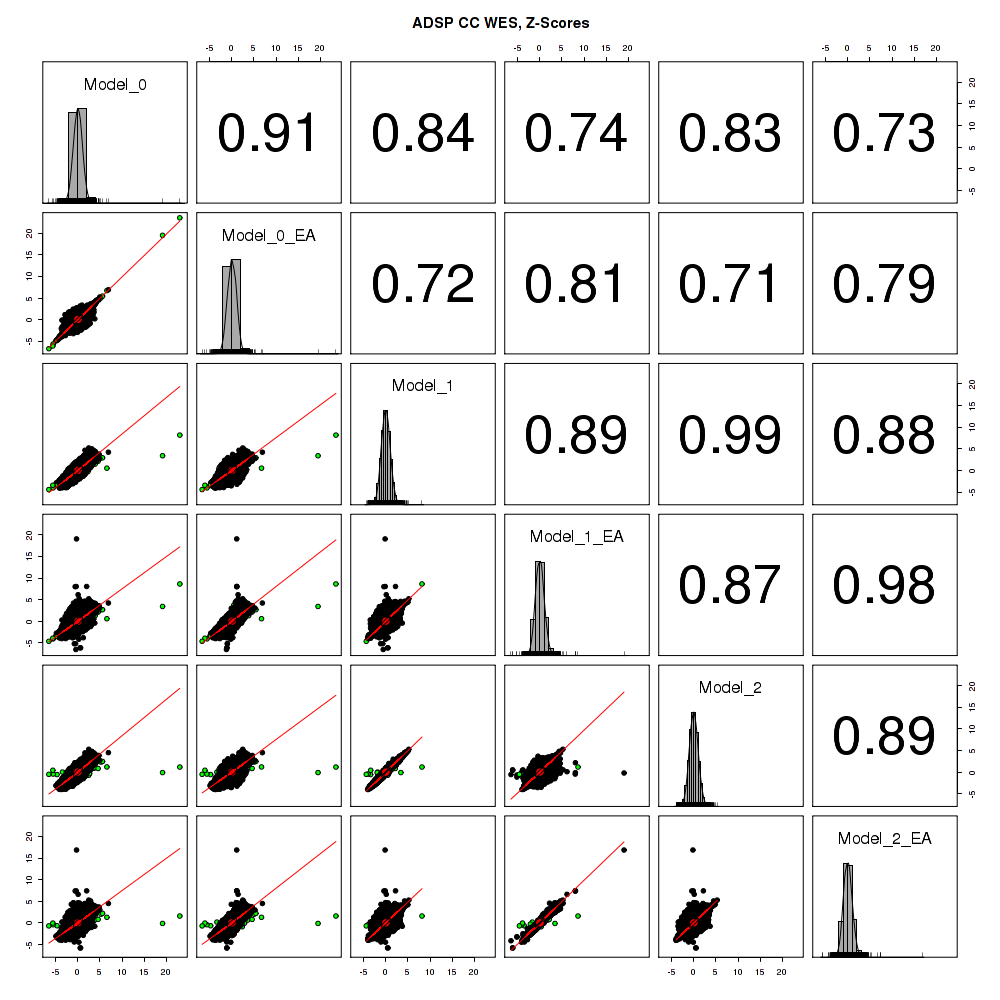** |

**Supplementary Figure S3.** Variant allele frequency by predicted impact


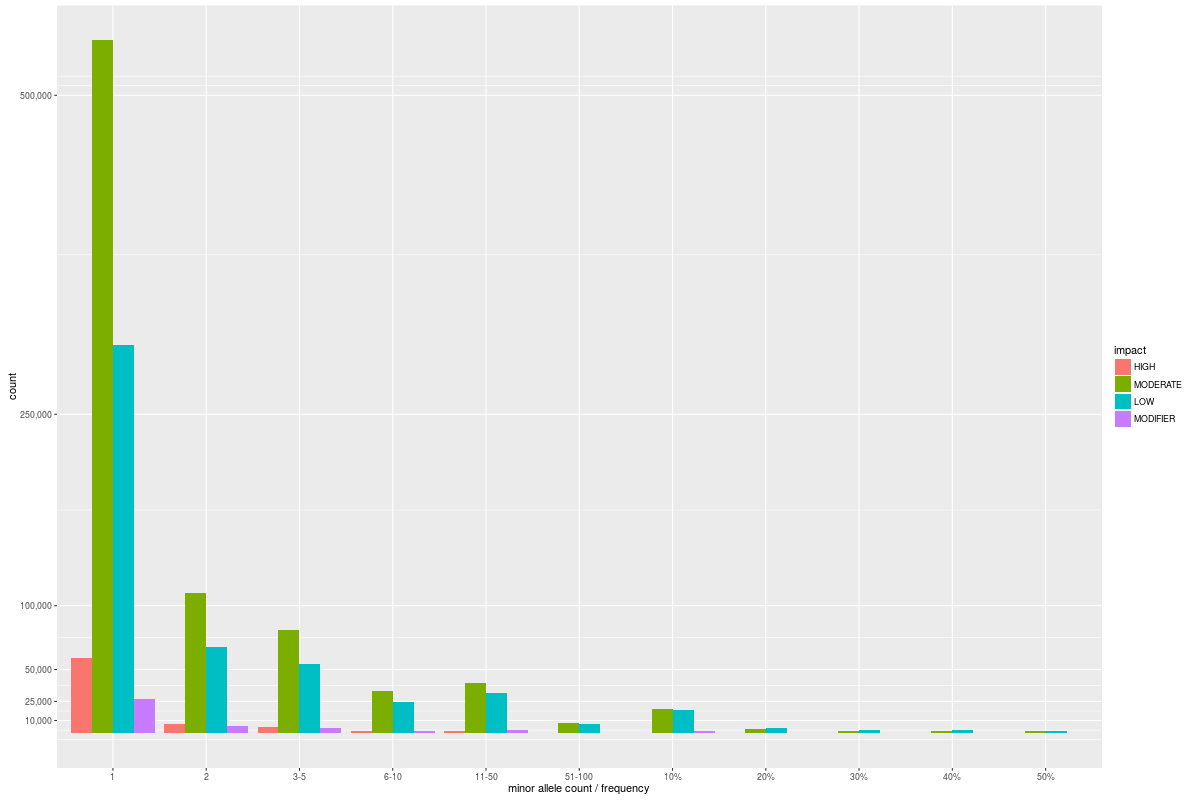


**Supplementary Figure S4**. QQ Plots for Individual Variants Meta Analyses. Plots show exome-wide Q-Q plots for each of the ADSP Discovery meta-analyses, stratified by minor allele count. Genomic inflation lambda values are shown separately by bins of minor allele count as well as for all variants.


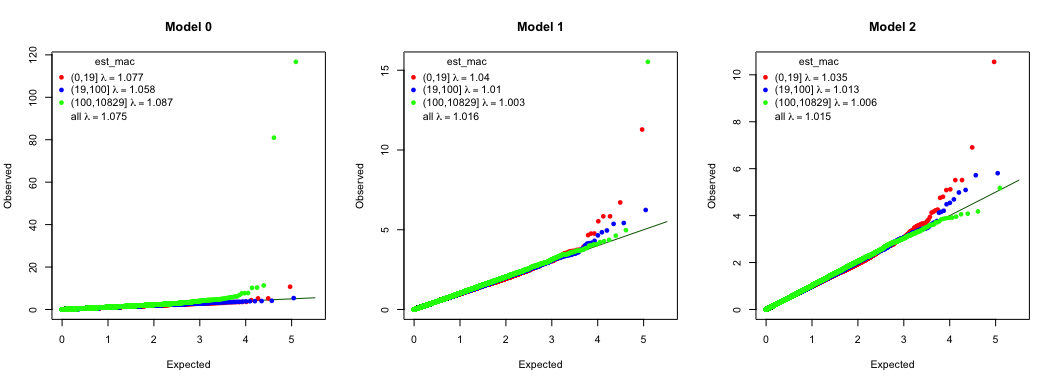


**Supplementary Figure S5.** QQ Plots for SKAT-O (moderate or high impact variants). Plots show exome-wide Q-Q plots for each of the ADSP Discovery meta-analyses, stratified by cumulative minor allele count across all high or moderate impact variants aggregated by gene. Genomic inflation lambda values are shown separately by bins of minor allele count as well as for all variants.


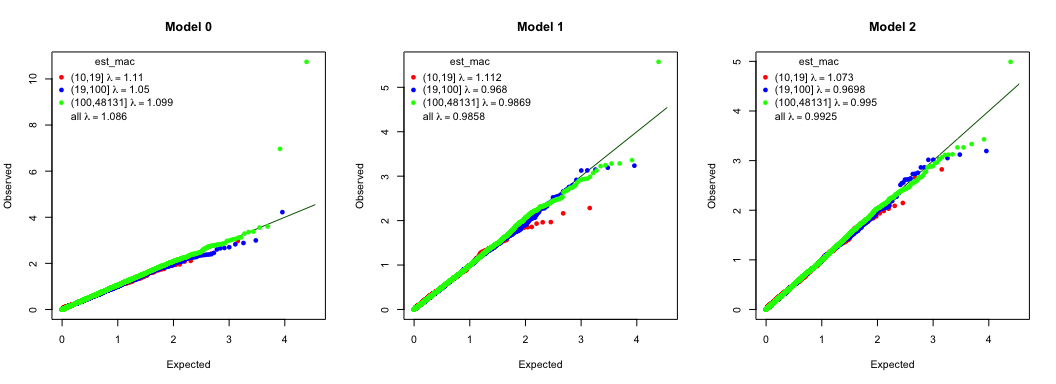


**Supplementary Figure S6.** QQ Plots for SKAT-O (high impact variants). Plots show exome-wide Q-Q plots for each of the ADSP Discovery meta-analyses, stratified by cumulative minor allele count across all high impact variants aggregated by gene. Lambda values are shown separately by bins of minor allele count as well as for all variants.


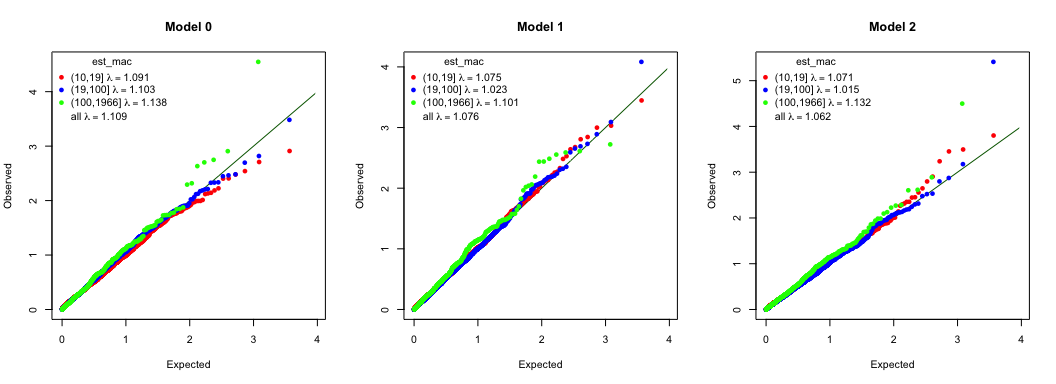


**Supplementary Figure S7.** QQ Plots for SKAT-O (loss-of-function variants). Plots show exome-wide Q-Q plots for each of the ADSP Discovery meta-analyses, stratified by cumulative minor allele count across all loss-of-function variants aggregated by gene. Lambda values are shown separately by bins of minor allele count as well as for all genes.

**
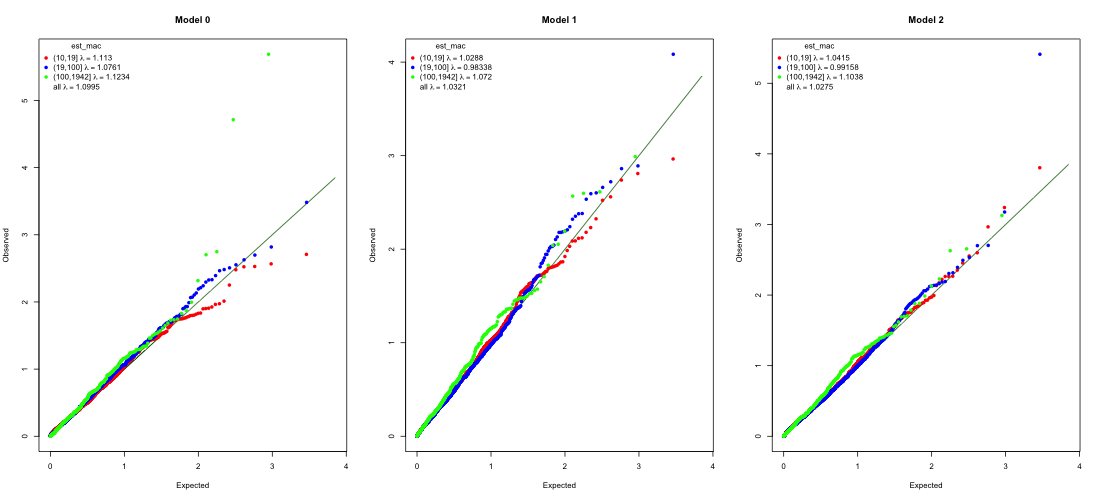
**

**Supplementary Figure S8.** Correlation of VEP annotation pipeline and CADD score results. **A.** VEP High/Moderate vs. CADD score > 15 results. **B.** VEP High vs. CADD score > 20 results. Genes with a cumulative minor allele frequency (cMAF) < 0.01 are shown in blue, genes with cMAF > 0.01 are shown in red.


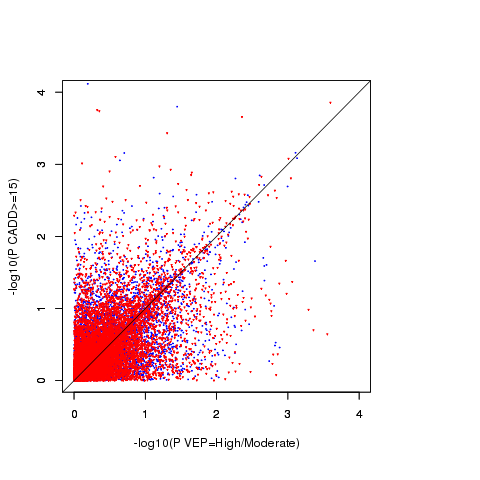

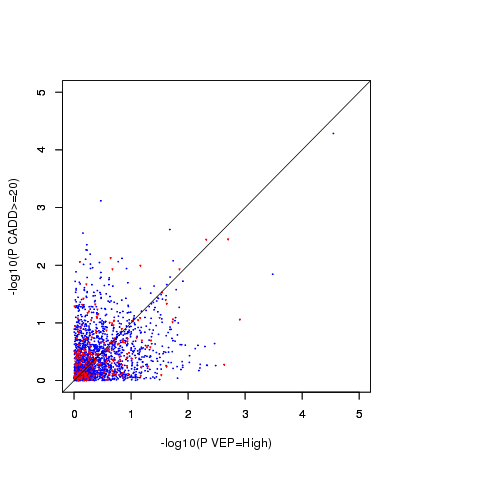


1. **B.**
